# Loss of dominant caterpillar genera in a protected tropical forest

**DOI:** 10.1038/s41598-019-57226-9

**Published:** 2020-01-16

**Authors:** Danielle M. Salcido, Matthew L. Forister, Humberto Garcia Lopez, Lee A. Dyer

**Affiliations:** Department of Biology, Program in Ecology, Evolution and Conservation Biology, University of Nevada, Reno, NV 89557 USA

**Keywords:** Ecology, Biodiversity, Climate-change ecology, Tropical ecology

## Abstract

Reports of biodiversity loss have increasingly focused on declines in abundance and diversity of insects, but it is still unclear if substantive insect diversity losses are occurring in intact low-latitude forests. We collected 22 years of plant-caterpillar-parasitoid data in a protected tropical forest and found reductions in the diversity and density of insects that appear to be partly driven by a changing climate and weather anomalies. Results also point to the potential influence of variables not directly measured in this study, including changes in land-use in nearby areas. We report a decline in parasitism that represents a reduction in an important ecosystem service: enemy control of primary consumers. The consequences of these changes are in many cases irreversible and are likely to be mirrored in nearby forests; overall declines in the region will have negative consequences for surrounding agriculture. The decline of important tropical taxa and associated ecosystem function illuminates the consequences of numerous threats to global insect diversity and provides additional impetus for research on tropical diversity.

## Introduction

The impacts of global change are multifaceted and ubiquitous^1^ with major ecological and evolutionary consequences^2^ that span aquatic and terrestrial ecosystems as well as a wide diversity of taxa and species interactions^3^. Much of global change research has focused on the negative consequences for single trophic levels, and despite an increased emphasis on interaction diversity in ecology^4^, relatively few studies have linked climatic variability to interaction diversity, ecosystem stability, and services of specific guilds, such as parasitoids. Past studies have also been geographically and taxonomically biased towards temperate ecosystems^5–8^ and the subset of tropical studies of global change tend to focus on vertebrates and focal tree species. Despite the fact that 85% of global insect diversity resides in the tropics^9^, current analyses on insect declines are primarily focused on western, higher-latitude regions: United States, Great Britain and Europe^10^. Thus, although it has been clear for some time that a sixth mass extinction event is underway^11^, only recently have studies attempted to document declines in insect diversity in intact tropical forests by quantifying abundances of species within common guilds^12^.

Documenting long term population trends and fluctuations in diversity in tropical insect communities is especially important because of an unjustified assumption that tropical communities are more stable^13,14^ and more resilient to multiple global change disruptions. Threats to insect diversity include climate change, habitat loss, fragmentation, invasive species, pesticides, and pollutants^15–20^, and the magnitude of these effects and associated levels of ecosystem resilience do indeed vary considerably across biogeographic regions. For example, changes in some climate parameters, such as mean annual temperature are most severe at the poles, and some of the most dramatic examples of biotic change have been observed at high latitudes, such as increased overwintering survival and voltinism in pest insects^21,22^. In contrast, increases in extreme weather events will likely have complex and large effects on lowland tropical communities, where plant-insect food webs may be particularly sensitive because of highly-specialized trophic relationships relative to interactions at higher latitudes^23^. Furthermore, vulnerability of tropical communities to global change is exacerbated by the thermal constraints of tropical ectotherms^24–26^, high degrees of endemism and high rates of tropical habitat loss^27–30^.

In general, reports on insect declines have mostly included cases where the causes are unspecified or unclear^12,31^, or the consequences to ecosystem services have not been explored^10,12,32^. Studies that span multiple decades and metanalyses examining a broad array of taxa have documented substantial reductions in insect abundance, biomass and diversity (for both temperate^32–38^ and tropical^12^ ecosystems). These changes in diversity have been associated with losses of rare species^33^ or increased dominance of generalists^36^. Putative mechanisms for these declines include habitat loss, conversion to arable land^33^, pesticide use^10,35^, increases in maximum temperature (T_max_)^12^, extreme weather events^35^, and synergisms among these factors^39^. Effect sizes reported in these studies are variable and suggest that the fate of insects will be determined by a complex mix of interacting stressors rather than any single cause^40^. The most thorough multi-decadal data for Lepidoptera show declines in abundance are widespread in temperate regions^37,41–43^, and these changes are due to a variety of global change parameters, for example loss of overwintering sites and degradation of breeding habitat for a migratory butterfly^44^. For associated loss in ecosystem services, insect declines have been linked to pollination services^45^, but to our knowledge an explicit connection between climate change and declines in parasitism has not been reported from long-term datasets.

**Figure 1:**
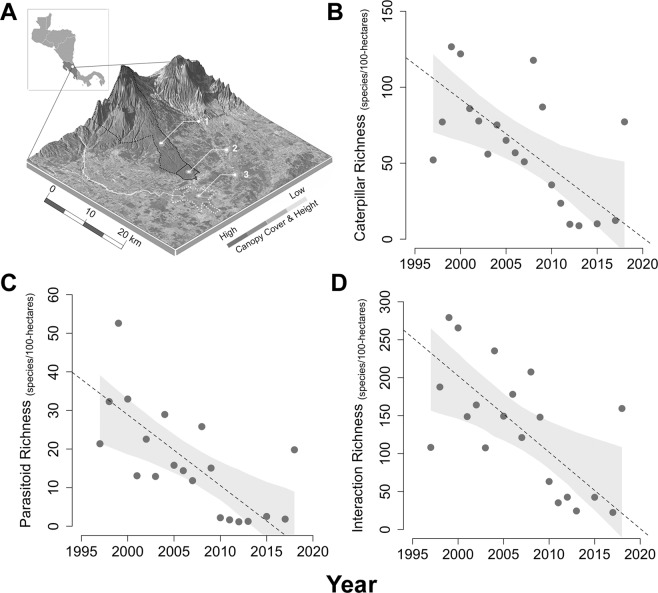
Caterpillar, parasitoid, and interaction richness declines across 22 years of sampling at La Selva Biological Research Station. Braulio Carillo National Forest (A.l) and surrounding areas, including La Selva (A.2) and a large adjacent banana plantation indicated by dashed white lines (A.3). Declines in caterpillar (**B**), associated parasitoid (**C**) and interaction (**D**) richness over the past 22 years (1997–2018) are evident within the La Selva forest patch. Dotted lines on plots depicting declines are the best fit lines from Bayesian regression, with 95% credible intervals in gray. Map designed by D.M.S.

Here, we contribute to understanding species declines and losses of biological interactions in a protected and well-studied tropical wet forest and examine potential losses of ecosystem function. The study area is La Selva Biological Research Station, Heredia Costa Rica (10° 26′ N, 83° 59′ W), a ~1600-hectare (ha) patch of forest on the eastern Caribbean slope of the Cordillera Central, bordered by agriculture as well as the Braulio Carrillio National Park (Fig. 1A). We used data from 1997 to 2018 to examine changes in taxonomic diversity among larval Lepidoptera (“caterpillars”) and associated parasitic Hymenoptera and Diptera (“parasitoids”).

## Results

### Ubiquitous declines in species richness across trophic levels

Our data reveal that declines in caterpillar and parasitoid richness (Fig. 1B,C) and diversity (Supplementary Fig. S1–S3) are widespread across the two consumer trophic levels (caterpillars: β = −0.03, 95% credible intervals (CI) [−0.06, −0.01], R^2^ = 0.43; parasitoids: β = −0.02, [−0.03,−0.01], R^2^ = 0.44). These coefficients represent a 9.48% and 14.76% decline in species per hectare each year for caterpillars and parasitoids, respectively. Extrapolation of estimated declines to the full 1600 ha of La Selva yielded estimates for the number of species that have either been lost from the forest since the start of the study or have been reduced to sufficiently low density that they are no longer detected (which likely amounts to effective extirpation from the perspective of ecological interactions): we estimate 1056 fewer caterpillar species (with 95% Bayesian credible intervals from 2112 to 352), and 704 fewer parasitoid species (from 1056 to 352). These are crude estimates of reduction based on numerous assumptions, including complete turnover per spatial unit (increasing the estimate of loss) and ignoring unquantified diversity (decreasing the estimate of loss). For the caterpillars, for which we have the most data, we additionally used the first 5 years of data to estimate a baseline diversity (Chao estimator) from which the losses represent an estimated 38.8% (with credible intervals from 77.6% to 12.9%).

### Loss of dominant caterpillar genera

**Figure 2:**
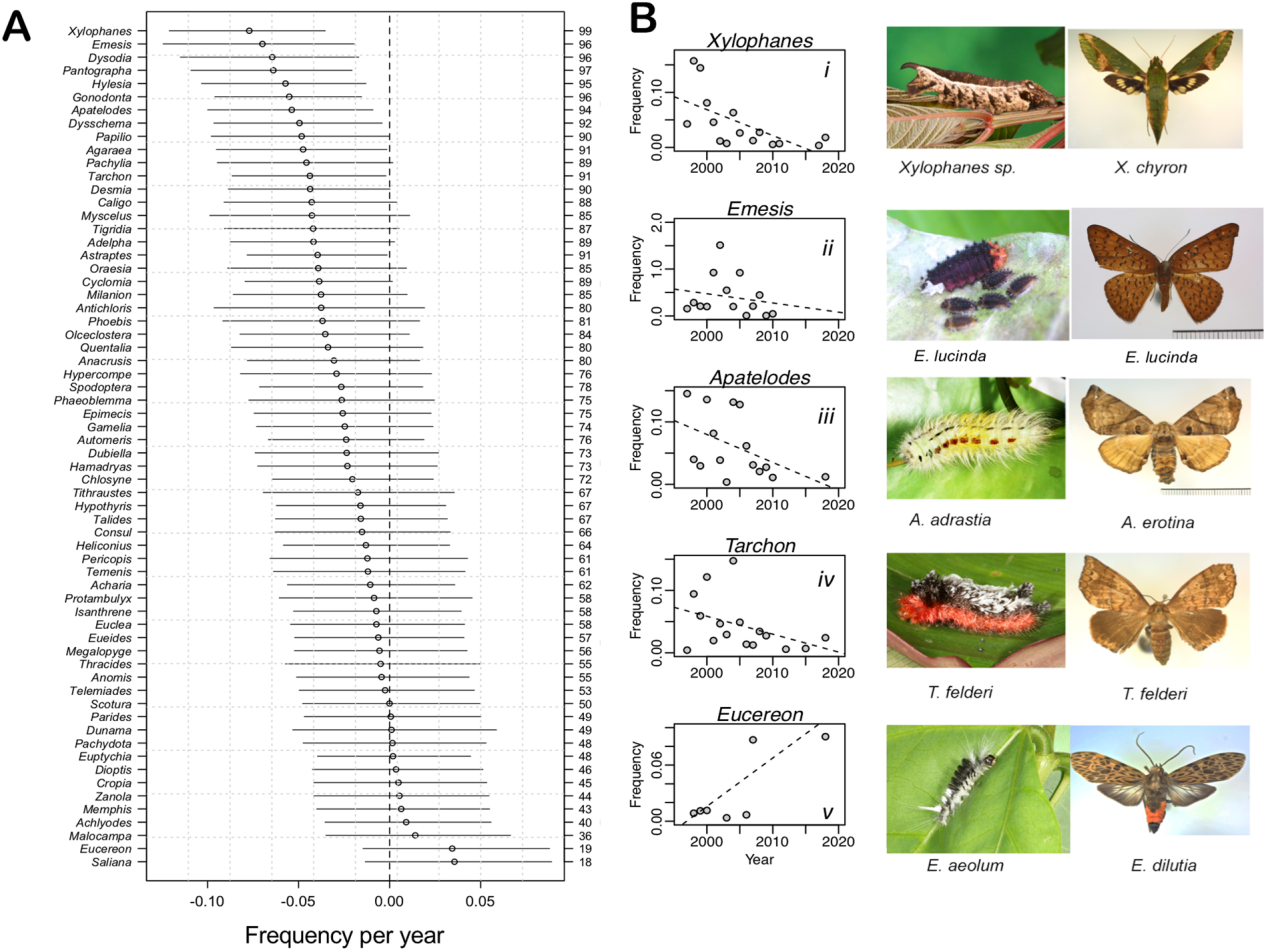
Genus-level patterns in caterpillar encounter frequencies across years. (**A**) Point estimates for beta coefficients and associated 80% credible intervals (CI) for 64 genera that comprise a subset of all genera collected that met criteria for this analysis. Genus names are listed on the left margin and probabilities of a decline are on the right margin. Units of the year coefficient are on the log-odds scale. (**B**) Frequency (untransformed) across years for select genera and representative larval and adult images. Photographs taken by L.A.D, D.M.S and H.G.L.

In addition to declines in caterpillar diversity, frequencies of encounter for entire genera of caterpillars are decreasing: out of the 64 genera studied, 41% (26 genera) have an 80% probability of being in decline (i.e. at least 80% of the mass of the Bayesian posterior distributions were less than zero for the year coefficients in regressions for each of these genera) (Fig. 2, Supplementary Table S1). Genera with greater than 95% probability of decline include: *Xylophanes* (99%), *Pantographa* (97%), *Emesis* (96%), *Dysodia* (96%), *Gonodonta* (96%) and *Hylesia* (95%). Across all genera studied, the decline in frequency across years estimated at the higher level of our hierarchical model is consistent with the picture of an overall decline (β = −0.13, 95% CI [−0.20, −0.05]; Supplementary Fig. S4) including a 99% probability that the beta coefficient across genera falls below zero. Declines for specific taxa were not affected by overall frequency of observation: less common species are not more likely to be in decline relative to more common species (Supplementary Fig. S5).

### Reduced ecosystem function as a consequence of declining parasitism frequency

**Figure 3:**
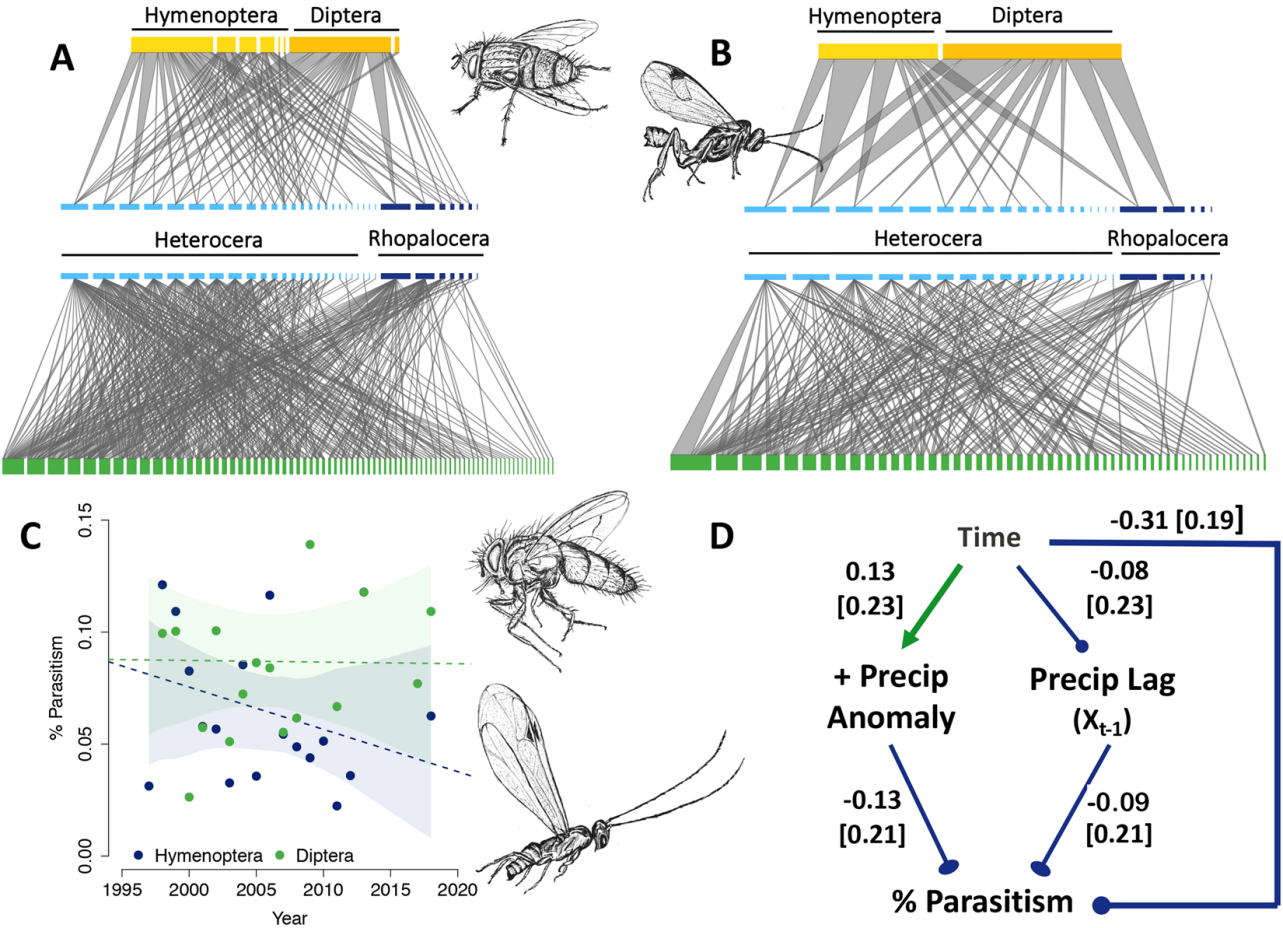
Patterns in plant-caterpillar-parasitoid interactions, climate, and parasitism across time. Tri-trophic networks illustrate host plants (green), caterpillars (blue), and associated parasitoids (yellow) for the first (**A**) and last 5 years (**B**) of the study. Nodes represent families within each trophic level and are grouped by caterpillar suborder (Heterocera: light blue and Rhopalocera: dark blue) and parasitoid order (Hymenoptera: light yellow and Diptera: mustard yellow) then ranked by node degree. Edge thickness represents relative link weights. Percent parasitism across the 22 years of the study period (**C**) for hymenopteran (blue) and dipteran (green) parasitoids. Structural equation model examining causal relationships among time, positive precipitation anomalies and their one-year time lag and parasitism (**D**). Path coefficients are standardized. Standard errors are reported in brackets. Arrows represent positive associations and lines with circles represent negative associations. The model is a good fit to the data: χ^2^ = 0.88, p = 0.35, df = 1, Parasitoid illustrations by M.L.F.

Along with taxonomic declines, interaction richness at La Selva is decreasing (β = −0.07, [−0.13, −0.02], R^2^ = 0.44; Fig. 1D): across the whole forest, assemblages today have approximately 2,464 fewer unique interactions (30.9% reduction) than networks of interactions 22 years ago (Fig. 3A,B, Supplementary Table S2 and S3). Caterpillar-parasitoid interactions were especially affected, with over 77% of connections disappearing between caterpillars and parasitoids when comparing networks of interactions in the first and last five years of the study. Losses in species and interaction diversity were paralleled by reductions in parasitism frequency, an important measure of natural biological control (β = −0.003, [−0.007, 0.001], R^2^ = 0.43; Fig. 3C & Supplementary Fig. S6). Estimates for declines in parasitism are equivalent to −3% per decade which represents a 6.6% decline during the study period. Further, the probability of a negative slope for overall parasitism across time was ~92%. Consistent with the hypotheses predicting the vulnerability of more specialized species to climate change^46^, declines in parasitism were greatest among hymenopteran parasitoids (β = −0.001 [−0.004,0.002], R^2^ = 0.44; Fig. 3C) compared to dipteran parasitoids (β = −0.00007 [−0.003, 0.003], R^2^ = 0.24; Fig. 3C). Overall, this represents a loss of 2.2% in parasitism by Hymenoptera compared to a loss in Diptera of 0.2% across the study period.

### Precipitation and temperature anomalies and means are increasing

Persistent changes in climate variables have occurred in this region (Supplementary Fig. S7–10, Table S4 and S5). Specifically, annual mean temperature (Supplementary Fig. S8A, Table S4) and precipitation (Supplementary Fig. S7A, Table S4) are both increasing at La Selva and trends with time vary across seasons (Supplementary Fig. S9–10, Table S5). In the last five years, the positive temperature anomalies is the greatest on record, with the greatest minimum temperature (T_min_) and maximum temperature anomalies occurring during the study period (Supplementary Fig. S8C,F,I). Precipitation anomalies are steadily increasing as well (Supplementary Fig. S7C).

### Precipitation anomalies and their one-year time lag caused declines in parasitism

Structural equation models (SEM) provided support for the hypothesis that climate averages and climate anomalies have negative effects on ecosystem function. Precipitation anomalies and their one-year time lag are among the most important factors causing lower parasitism frequency (Fig. 3D; χ^2^ = 0.88, p = 0.35, df = 1; p > 0.05 indicates fit of the model to the data). Specifically, precipitation anomalies had a significant negative effect on percent parasitism (standardized path coefficient (hereafter, spc) = −0.13) as did the one-year time lags of extreme precipitation events (spc = −0.09).

### Temperature and precipitation anomalies and averages caused declines in species and interaction richness

**Figure 4:**
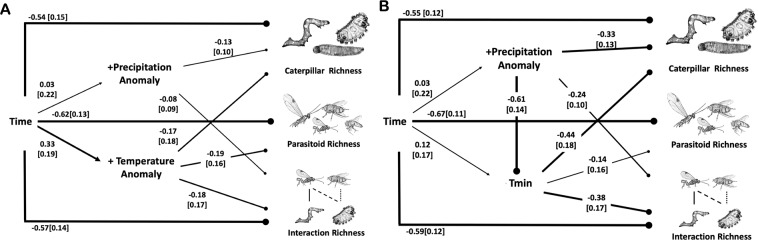
Structural equation models (SEM) estimating the effects of climate variables on caterpillar, parasitoid and interaction richness. (**A**) Associations between time (year), richness, positive temperature anomalies and precipitation anomalies; model fit: χ^2^ = 0.16, p = 0.91, df = 2. (**B**) Similar causal pathways as in the previous panel, but with average daily minimum temperatures in place of temperature anomalies; this model also controls for the indirect effect of precipitation anomalies on richness mediated through effects on minimum temperature (which decrease with more flooding); model fit: *χ*^2^: 0.02, p = 0.89, df = 1. Path coefficients are standardized and width of arrows are scaled based on magnitude of path coefficients. Arrows represent positive associations and lines with circle represent negative associations. Parasitoid illustrations by M.L.F. Caterpillar images by B.L.

Declines in richness are also linked to changes in a number of climate variables. The best fit models provide support for the inference that declines in richness are caused by increases in precipitation and temperature anomalies (χ^2^ = 0.16, p = 0.91, df = 2, Fig. 4A) and by increases in positive precipitation anomalies and minimum (*χ*^2^ = 0.02, p = 0.89, df = 1, Fig. 4B) or maximum (*χ*^2^ = 0.27, p = 0.87, df = 2, Fig. S11) temperatures. For models testing relationships among precipitation anomalies and temperature variables, the most parsimonious models excluded small associations among precipitation anomalies and parasitoid richness. Temperature anomalies had large negative effects on all levels of richness (spc_caterpillar_ = −0.17, spc_parasitoid_ = −0.19, spc_interaction_ = −0.18, Fig. 4A), and precipitation anomalies had more subtle negative effects on caterpillar and interaction richness (spc_caterpillar_ = −0.13, spc_interaction_ = −0.08, Fig. 4A). Rising minimum temperatures (spc = 0.12, Fig. 4B) have caused relatively large decreases in all levels of richness (spc_caterpillar_ = −0.44, spc_parasitoid_ = −0.14, spc_interaction_ = −0.38) and the direct negative effects of precipitation anomalies on richness (spc_caterpillar_ = −0.33, spc_interaction_ = −0.24) increase by an order of magnitude when controlling for associations with minimum temperature (spc = −0.61). Rising maximum temperatures (spc = 0.60, Fig. S11) have also caused relatively large decreases in all levels of richness (spc_caterpillar_ = −0.24, spc_parasitoid_ = −0.18, spc_interaction_ = −0.19). Precipitation time-lags had variable effects on richness compared to present year anomalies (Supplementary Fig. S11).

## Discussion

The dramatic declines reported here suggest that many caterpillars at La Selva will be losers and few will be winners in response to global change^47^, resulting in an overall reduction in the role of caterpillars as herbivores and as food for other animals. Compelling examples of winners and losers include the success of the genus *Eucereon*, which includes outbreak species^48^, and the failure of formerly common genera, such as *Emesis* (Fig. 2 & Supplementary Fig. S5). The observed loss of common insect taxa may have cascading effects on many other organisms at La Selva; declines in insectivorous vertebrate predators, including bats and birds within and near the forest, have already been attributed to reductions in arthropod prey^49,50^. Not only are these insects important prey, but the insects that are declining at La Selva are involved in numerous ecosystem processes, including parasitism, pollination, and plant consumption. In general, declines in caterpillars indicate an overall decline in environmental suitability, as butterflies and other moth taxa are considered indicator taxa^51^.

As we have documented at La Selva, one consequence of species extirpation or declines is the loss of interspecific interactions, which are the basis of ecosystem stability and ecosystem services^52^. Questions about loss of interaction diversity are largely absent from global change literature, due largely to a dearth of quantitative empirical data^53–55^. Reductions in species^56^ and interaction diversity^57^ can cause reduced ecosystem function via loss of functional redundancy, with likely cascading effects on natural biological control, pollination, plant diversity, primary productivity, and nutrient cycling. The declines in parasitism reported here can be extrapolated to an impressive 30% drop in parasitism over the next 100 years, which is a major loss of a key ecosystem service that prevents damaging outbreaks of herbivorous insects^58^. Losses of species and trophic interactions of this magnitude are particularly relevant in areas with intensified agriculture, where the global economic contribution of biological control is now estimated at $1.56 trillion per year^58,59^. The tropics currently have the greatest rates of agricultural expansion and tropical agriculture is expected to expand by at least 50% by 2050^60,61^. For the continually expanding agricultural areas surrounding La Selva, parasitoids are essential for biological control; for example parasitoids are an effective control of herbivores for banana crops^62^, and over 10,000 ha of land surrounding La Selva are banana plantations with one of the largest plantations situated less than 3 km from La Selva (Fig. 1A.3).

Climate-driven declines in parasitism were paralleled by climate-driven declines in caterpillar and parasitoid species and interaction richness; this relationship between parasitism and precipitation anomalies corroborated predictions in Stireman *et al*^46^. Anomalies in precipitation (extreme wet events) and temperature (warmer than average episodes) are occurring at an increasing rate at La Selva, which is consistent with the idea that tropical ecosystems are facing increasing climatic variability and extremes^63,64^. Among models that included causal links between climate variables and diversity (species and interaction richness), increases in temperature and precipitation anomalies negatively affected caterpillar and parasitoid richness and associated interactions in our study area. More variable precipitation and temperature, including an increase in extreme weather events, can lead to the observed community-level changes in myriad ways, especially with respect to changes in biotic interactions^3,65^. For example, phenological asynchrony can lead to local extinction or alter spatial and temporal turnover in species and interactions. Further, local extirpation of host populations or reductions in their size from flooding or drought events could have caused the observed declines in parasitoid diversity over long periods of time. These possibilities will wait for further study, as our current analyses were not designed to distinguish among them.

In addition to an elevated frequency of weather anomalies, minimum and maximum daily temperatures are increasing and appear to have a negative effect on the observed richness of caterpillars and interactions. Although it is unclear if tropical ectotherms will be more sensitive to changes in temperature relative to higher-latitude species^66^, our results confirm that tropical insect food webs are affected by warming conditions. Increases in minimum temperature are consistent with responses reported in temperate butterfly systems^67^. Interspecific variation in thermal optima is high^68^, and physiological responses to increases in minimum and maximum temperatures are likely to be quite variable. To attenuate the physiological effects of increases in daily temperature extremes, insects may constrain or modify host search activity, which can affect community composition and structure through changes in interaction strength, symmetry, and turnover over across time and space^69^.

In all models, time had the strongest (direct negative) effect on richness and parasitism frequency compared to other predictors, suggesting that other unmeasured global change variables also contribute to the observed declines at La Selva, such as habitat loss, agricultural intensification and exotic species introductions. Between 1990 and 2010, forest cover in Central and South America declined by 56.9 million hectares^70^ (a rate equivalent to ~1613 La Selva forest patches deforested each year) and protected remnants have become increasingly isolated as forested buffers disappear^71^. La Selva and the surrounding areas are no exception. Seventy-eight percent of the tropical wet forest in Costa Rica was deforested over the past 50 years, the majority of which was converted to pasture^72^. While the annual deforestation rate in the Sarapiquí canton declined to 6.7% during the course of our study^30^, the rapid conversion of pasture to high-yield, high input plantation farming tripled^73^ and pesticide use increased substantially^74^. In Costa Rica, rates of agricultural pesticide application rank second globally compared to other countries^75^ at 18.78 kg/ha, and the amounts applied to export crops farmed in Sarapiquí such as banana (49.3 kg a.i/ha) and pineapple (25.2 kg a.i./ha) exceeds this average^74^. The extent and consequences of pesticide drift on ecological assemblages within La Selva are unknown, but negative effects on parasitoids have been documented in agricultural systems in the region^62,76^. Pesticides can reduce species and interaction diversity through direct effects on herbivore and natural enemy abundance and via indirect effects on species interactions, for example via reduced herbivore immune response^77^. In addition, disturbances (in the form of development or habitat conversion) and species introductions may have altered host plant richness and contributed to insect declines. Species introduced to La Selva include *Erythrina poeppigiana* (Fabaceae), *Musa velutina* (Musaceae), *Elaeis guineensis* (Arecaceae) and *Theobroma cacao (Malvaceae*). Potentially, these invasive species could reduce caterpillar diversity through competitive exclusion of host plants (but see^78^).

Declines in populations of plants and animals, extinctions, and associated loss of ecosystem function are defining features of the Anthropocene^11^. From a general Bayesian perspective in which new results are used to update prior knowledge^79^, additional corroborations of these Anthropocene-associated losses are useful in that they provide more precise estimates of decline probability for specific taxa, regions and ecosystems. Although insect declines have been the subject of recent high-profile studies^10,12,32^, the taxonomic and geographic breadth of the phenomenon is not without controversy^80^ and reports have been rare from the planet’s most species-rich ecosystems. Thus, we suggest that the results reported here strengthen the growing probability that insects are facing what indeed may be a global crisis. The hard work that still faces ecologists is to try to figure out which traits and habitats most expose species to risk, while the challenges for taxonomists and natural historians are to discover and describe new species and interactions before they disappear. All biologists should be considering how to use existing data to focus on the most imperiled taxa, ecosystems, and biogeographic regions. Tropical wet forests are clearly one biome requiring more precise estimates of species declines and a better understanding of determinants of these declines. For La Selva, our results are consistent with the hypothesis that climate change in lowland tropical forests is contributing to declines in species and entire genera of caterpillars as well specialized parasitoids. Although such multi-trophic connections are not frequently studied in the context of global change, if results such as ours are widespread, then cascading results to other guilds and trophic levels can be expected^52^ and warrant immediate concern and management effort.

## Methods

### Study sites and sample methods

We collected plant-caterpillar-parasitoid interaction data within La Selva across all seasons. Seasonality is marked by a wet season from May to December and a brief dry season from January to April; peak rainfall occurs in June-July, and March is the peak dry month. Samples were collected as a larger rearing program cataloguing plant-caterpillar-parasitoid associations across the Americas^81,82^ from 1995 to present. We limited our results to records starting in 1997 up to 2018, and we excluded 2014 and 2016 because sampling days did not meet our minimum criteria of 20 sampling days/year. We sampled externally feeding (including shelter builders) caterpillars from their host plants and reared them to adult moths or parasitoids. Caterpillars were located opportunistically by visual inspection along trail transects (distance varies between 50 and 3000-meter and select transects were continuously sampled across years), or exhaustively sampled in 10 m diameter plots (149 plots total) by staff scientists, graduate students, parataxonomists, and teams of Earthwatch volunteers and students. Due to variable sampling days across years, we weighted observed values by sampling effort. Sampling effort was calculated as the number of volunteer and staff days of sampling multiplied by the average area in square meters (m^2^) covered by each person in a 10-day sampling period (4000 m^2^); hence, observed diversity and frequency is analyzed in models as species equivalents or frequencies per hectare per year (ha^−1^year^−1^). Diversity is presented as species equivalents per 100 ha^−1^year^−1^. We excluded *Eois* (Geometridae) and *Quadrus* (Hesperiidae) from all analyses because these focal genera present a bias in the rearing dataset due to focused collection for ancillary studies. We include a summary of total samples collected per trophic level per year and annual sampling effort in the supplemental information (Supplementary Table S6).

### Rearing methods & processing data

For our ongoing interaction diversity survey, collected larvae are given a unique voucher code that associates them with their host plant species. Caterpillars are reared individually in plastic containers or bags with a sample of host plant. Species identifications are made initially by parataxonomists to lowest taxonomic level or morphospecies and verified by taxonomic experts or by referencing voucher specimens and image libraries. Some morphospecies are confirmed using mtDNA COI sequences, others by examining a mix of morphological characters, and others using genomic data. For the remaining species without morphospecies designations, we assign morphotypes based on feeding relationships – morphologically distinct caterpillars from the same family utilizing the same host family are designated a unique morphotype. This method is likely a conservative means to assigning species names, especially for tropical species^23,82^. Voucher specimens are sent to collaborating institutions including universities and museums (see www. caterpillars.org for a list of participating institutions).

### Patterns in diversity, parasitism, climate variables

#### Abundance & diversity

We quantified species abundances and unique interaction frequencies for each year to compare patterns in diversity and abundance across time. We also aggregated the data to examine annual genus-level frequencies of caterpillars to evaluate declines of higher taxa; genus-level abundance data analyses only included genera with ≥ 5 years of data and sampling that extended to 2010. Results are reported for the 64 caterpillar genera that met these criteria. To obtain values of interaction diversity, we modified a community matrix such that rows were comprised of years and columns the unique interactions. Interactions were comprised of bi-trophic (plant-caterpillar) and tri-trophic (plant-caterpillar-parasitoid) interactions and each matrix cell represented annual frequencies of those interactions.

Diversity was calculated using Hill numbers, and values were interpreted as interaction or species equivalents^83,84^. Hill numbers vary as a function of the parameter q and indicate the sensitivity of the index to rare species, and q = 0, q = 1 and q = 2 are equivalent to species richness, Shannon’s diversity, and Simpson’s diversity, respectively. We used functions provided in Chao *et al*.^84^ to calculate Hill numbers. Results for q = 0 are reported in the main text and q = 1 and 2 in the supplemental information (see Supplementary Fig. S1–3). To obtain estimated percent caterpillar loss we quantified mean (Chao estimated) diversity for the first 5 years of data and subtracted species decline estimated from beta coefficients of the linear models of diversity across years.

#### Climate variables

Climate variables were calculated as annual means of daily precipitation and average, minimum (T_min_) and maximum (T_max_) temperatures. We used meteorological data acquired from weather stations within La Selva from 1983 to 2018^85^. Temperature is reported as degrees Celsius (°C) and precipitation in millimeters (mm). To examine effects of extreme weather events and climate variability on patterns of diversity, we calculated anomalies and the coefficients of variation (CV) for each precipitation and temperature variable. Precipitation anomalies were calculated as the sum of daily values exceeding 2.5 standard deviations (sd) of the annual mean. Similarly, for temperature anomalies we used 2sd. The coefficient of variation was calculated as the ratio of standard deviation to the annual mean. We used simple linear regression to evaluate patterns among each climate variable across time (Supplementary Fig. S7–8) and with respect to each season in the supplemental figures (Supplementary Fig. S9–10).

#### Evaluating patterns in network structural properties

We pooled interaction data to the family level for the first (1997–2001) and last (2012–2018) five years of collection to illustrate changes in tri-trophic network structure. For each network we calculated node degrees and relative edge weights and reported link and node richness for each trophic level (Supplementary Table S2).

#### Parasitism frequency

Percent parasitism was calculated as the ratio of parasitism events to all successfully emerged adults (caterpillars plus parasitoids) for each month from 1997 to 2018. We examined monthly trends across time to account for intra-annual and seasonal variation in tropical population dynamics. Excluded from analyses were months with zero parasitism or zero eclosed caterpillars as well as months without a number of adults that exceeded the 1^st^ quantile (Q_1_) of the distribution of total adults (IQR = 12–103).

### Statistical models

#### Bayesian models

We used Bayesian linear models to estimate coefficients for change over time for richness and diversity of caterpillars, parasitoids, and interactions, as well as parasitism frequency. Models were fit for total parasitism and separately for specialized (Hymenoptera) and non-specialized (Diptera) orders of parasitoids. Models were fit in JAGS (version 3.2.0) utilizing the rjags package in R^86^ using (for each analysis) two Markov chains and 1,000,000 steps each; performance was assessed through examination of chain histories (burnin was not required), effective sample sizes and the Gelman and Rubin convergence diagnostic^87^. Response variables were modeled as normal distributions with means dependent on an intercept plus predictor variables (either year alone, or year plus climatic variables), and we used uninformative priors: priors on beta coefficients (for year and climatic variables) were normal distributions with mean of zero and precision of 0.001 (variance = 1000); priors on precisions were modeled as gamma distributions with rate = 0.1 and shape = 0.1. All data was z transformed prior to analysis.

An additional hierarchical model (with uninformative priors as already described) was used to estimate change across years in the frequency of observations of individual caterpillar genera, with the year coefficients (and intercepts) estimated for each genus separately (as the lower level in the hierarchy) and simultaneously across all genera (the response variable for this analysis was log-transformed prior to z-transformation). For all models (simple and hierarchical) we retained point estimates from posterior distributions for beta coefficients, as well as 95% credible intervals (CI) for the diversity models. For the hierarchical model, we report 80% credible intervals for each genus but use 95% CI across all genera. We used the more liberal calculation of intervals for the former in the interest of minimizing incorrect inferences that declines of entire genera are unlikely (i.e. negative slopes have a low probability). We would rather risk the possibility of erroneously inferring decline as opposed to mistakenly concluding that a declining taxon is stable. As a complementary measure of confidence not associated with an arbitrary cutoff for importance, we calculated (for the beta coefficients estimated for each genus) the fraction of the posterior distribution less than zero, which can be interpreted as the probability that a genus has been observed with decreasing frequency over time. For Bayesian models, we calculated R-squared values following Gelman *et al*.^88^, with the exception of the model on genus-level declines where the hierarchy makes a single coefficient of determination less relevant.

#### Structural equation models

We used Structural Equation Modeling (SEM) to test causal hypotheses that evaluated the effect of climate and time on taxonomic and interaction richness and parasitism. We used the global estimation method in the lavaan package v.0.6–3^89^ in R v 3.5.3 to generate 5 models. The first model tested causal relationships among time, positive temperature and precipitation anomalies, and species and interaction richness (Fig. 4A). Similarly, a second and third model examined causal relationships among minimum temperature (Fig. 4B) or maximum temperature (Supplementary Fig. S11) and richness variables. For the fourth model, following a priori expectations of relationships between climate variables and parasitism^46^ we examined causal hypotheses that parasitism levels are determined by precipitation and its one-year lag (Fig. 3D). Finally, we examined the effects of time lags on species and interaction richness as well (Supplementary Fig. S12). Model fit was assessed using χ2 values and models were compared using Akaike information criteria (AIC). We reported standardized path coefficients and illustrated the SEM results in a path diagram.

## Supplementary information

Supplementary information.

## Data Availability

Should the manuscript be accepted, the data supporting the results will be archived in an appropriate public repository and the data DOI will be included at the end of the article. All data needed to evaluate the conclusions in the paper are available upon request.
