# Supplementary material for: Loss of dominant caterpillar genera in a protected tropical forest
Source: Sci Rep. 2020 Jan 16;10:422. doi: 10.1038/s41598-019-57226-9 (PMC6965627; doi:10.1038/s41598-019-57226-9)
Supplement: Supplementary file 1 — Supplementary information. [file 41598_2019_57226_MOESM1_ESM.docx]

**SUPPORTING INFORMATION**

**Supplementary Fig. S1.** Lepidoptera diversity measured as effective number of species per hectare across sampling years. Each panel represents Hill numbers between 0 and 2. Beta coefficients were estimated using Bayesian linear models and shaded areas represent 95% credible intervals.

**Supplementary Fig. S2.** Parasitoid diversity measured as effective number of species per hectare across sampling years. Each panel represents Hill numbers between 0 and 2. Beta coefficients were estimated using Bayesian linear models and shaded areas represent 95% credible intervals.

**Supplementary Fig. S3.** Tri-trophic interaction diversity measured as effective number of interactions per hectare across sampling years. Each panel represents Hill numbers between 0 and 2. Beta coefficients were estimated using Bayesian linear models and shaded areas represent 95% credible intervals.

**Supplementary Fig S4.** Posterior distributions for beta coefficients from models examining the frequency of observations of caterpillar genera across years. Distributions for each of the 64 caterpillar genera (red distributions) and across all (black distribution) are included. The 50% quantile (solid red line; β= -0.13), the 90% credible intervals (CI) (dashed red lines; [-0.19, -0.06], and 95% (CI) (dashed blue lines; [-0.20, -0.05]) are indicated for the posterior distribution. Across all genera, 99% of the mass of the posterior distribution falls below zero (black dashed line).

**Supplementary Fig. S5.** Beta coefficients for years plotted against raw frequencies of observation for 64 Lepidoptera genera. The blue points the two biggest ‘winners’ (*Saliana (A) and Euceron* (B)) and the red points are our two biggest ‘losers’ (*Xylophanes (C) and Emesis* (D)).

**Supplementary Fig. S6.** Parasitism frequency at La Selva across years of study (1997-2018). A Bayesian linear model was used to estimate beta coefficient (β =-0.003, [-0.007,0.001]); the shaded area displays 95% credible intervals.

**Supplementary Fig. S7.** Patterns in precipitation variables at La Selva from 1982 to 2018. Each point represents a year of data. Precip (mm) is the annual mean of daily precipitation, the coefficient of variation (CV) in precipitation is intra-annual CV, days of extreme precipitation are counts of daily precipitation exceeding 2.5 SD of annual mean precipitation, and dry days were calculated as total days within a year with zero rainfall.

**Supplementary Fig. S8.** Patterns in temperature variables (average, minimum and maximum daily temperature) at La Selva from 1982-2018. Each point represents a year of data. Graphs in the first row represent annual means of daily values for each temperature variable. The second row displays the intra-annual coefficient of variation. The third row displays temperature anomalies measured as the sum of daily values exceeding 2 standard deviations of the annual mean.

**Supplementary Fig. S9.**  Patterns in temperature variables at La Selva from 1982 to 2018 for the wet (blue) and dry (red) season. The wet season includes data from May to December and results for the dry season include January-April. Each point represents a year of data. Graphs in the first row represent annual means of daily values for each temperature variable. The second row displays the intra-annual coefficient of variation. The third row displays temperature anomalies measured as the sum of daily values exceeding 2 standard deviations of the annual mean.

**Supplementary Fig. S10.**  Patterns in precipitation variables at La Selva from 1982 to 2018 for the wet (blue) and dry (red) season. The wet season includes data from May-December and results for the dry season include January-April. Precip (mm) is calculated as the annual mean of daily precipitation, the Coefficient of Variation (CV) in precipitation is calculated as intra-annual CV, days of extreme precipitation are counts of daily precipitation exceeding 2.5 standard deviations of annual mean precipitation, and dry days are calculated as total days within a year with zero rainfall.

**Supplementary Fig. S11.** Structural equation models (SEM) testing the effects of maximum temperature (T_max_), and precipitation anomalies on caterpillar, parasitoid and interaction richness. Time is an exogenous variable representing year, and the endogenous variables include richness, T_max_, and positive precipitation anomalies; model fit: *χ*^2^ =0.27, p=0.87, df=2. Path coefficients are standardized and width of arrows are scaled based on magnitude of path coefficients. Standard errors are reported in brackets. Arrows represent positive associations and lines with circle represent negative associations. Parasitoid illustrations by M.L.F. Caterpillar images by B.L.

**Supplementary Fig. S12.** Structural equation models (SEM) testing the effects of positive temperature anomalies and their one year time lag (x_t-1_) on caterpillar, parasitoid and interaction richness. Time is an exogenous variable representing year and the endogenous variables include richness, precipitation anomalies, and time lags; model fit: χ^2^ =0.78, p= 0.67, df=2. Path coefficients are standardized and width of arrows are scaled based on magnitude of path coefficients. Standard errors are reported in brackets. Arrows represent positive associations and lines with circle represent negative associations. Parasitoid illustrations by M.L.F. Caterpillar images by B.L.

**Supplementary Table S1.** Estimates of standardized and unstandardized beta coefficients and associated 80% credible intervals for each Lepidoptera genera nested in a hierarchical Bayesian model that modeled frequency across years.

**Supplementary Table S2.** Quantitative comparisons among observed network metrics summed for the first (1997-2001) and last (2012-2018) five years of data. Values represent network properties summarized at the level of taxonomic families, and (in square brackets) at the level of species.

**Supplementary Table S3.** Interaction turnover (β_wn_) and its components among plant-herbivore and herbivore-parasitoid species-level interactions for networks representing the first (1997-2001) and last (2012-2018) five years of data. Interaction turnover among two networks is the sum of turnover owed to differences in species composition (β_ST_) and shared species interacting differently (β_OS_). Species turnover (β_S_) is included for reference.

**Supplementary Table S4.** Linear model estimates and fit for various climate variables collected from 1983 to 2018.

**Supplementary Table S5**. Linear model estimates and fit for various climate variables collected from 1983 to 2018. Data is displayed for wet (May-December) and dry (January-April) seasons separately.

**Supplementary Table S6**. Summary statistics describing annual sampling totals across plant, caterpillar and parasitoid taxonomical groupings.

**Supplementary Figure S1**


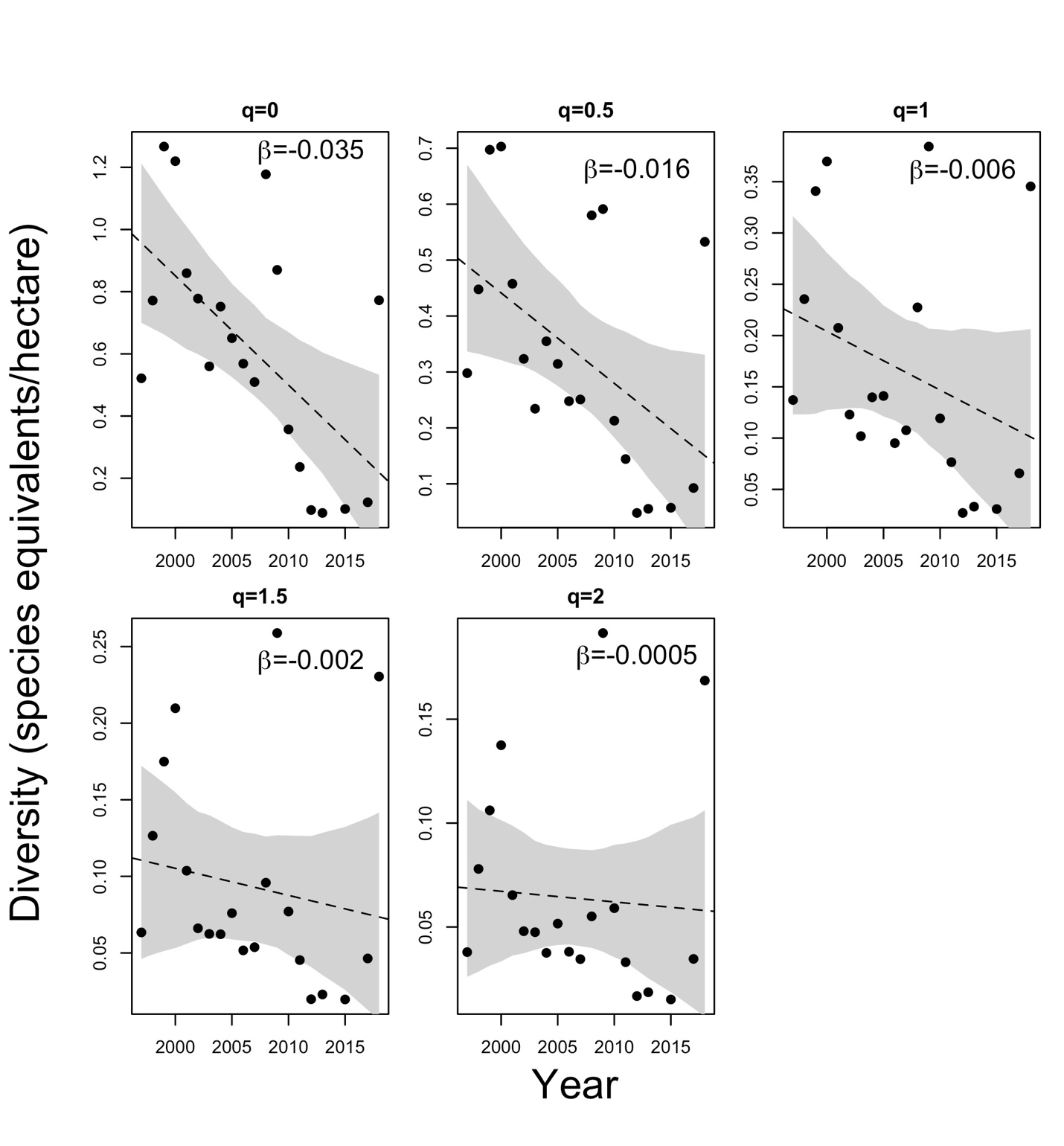


**Supplementary Figure S2**


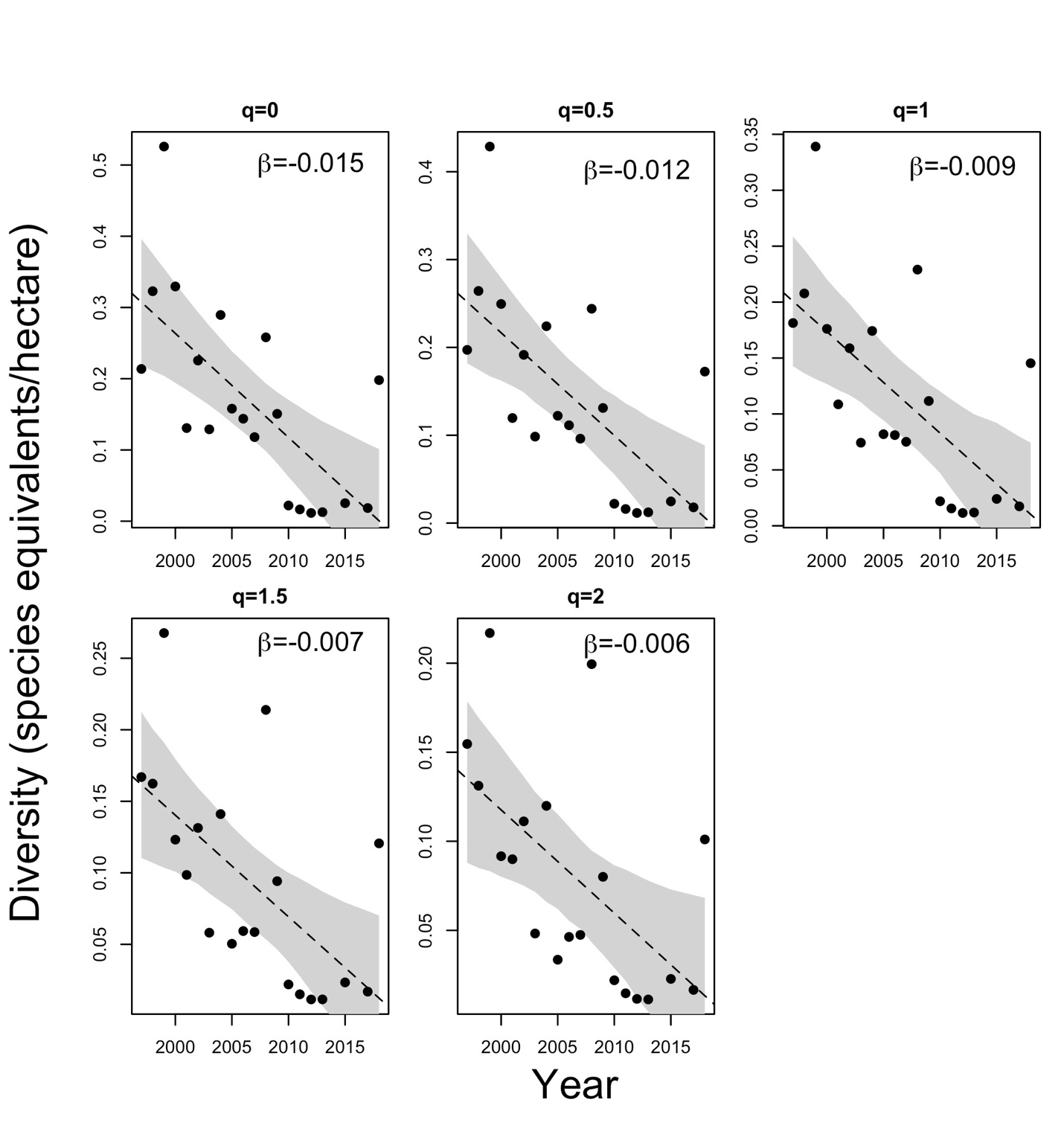


**Supplementary Figure S3.**

**
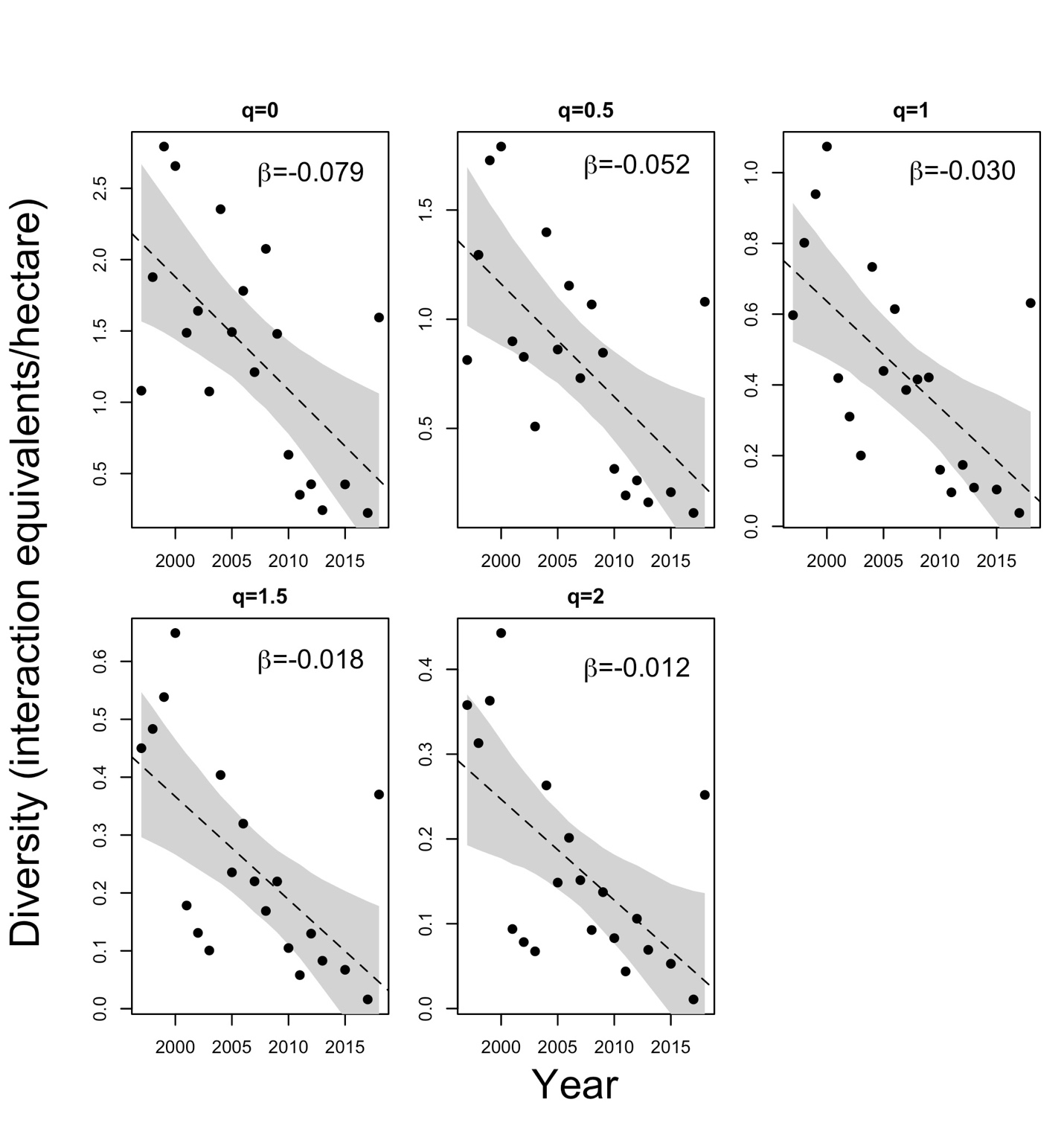
**

**Supplementary Figure S4.**

**
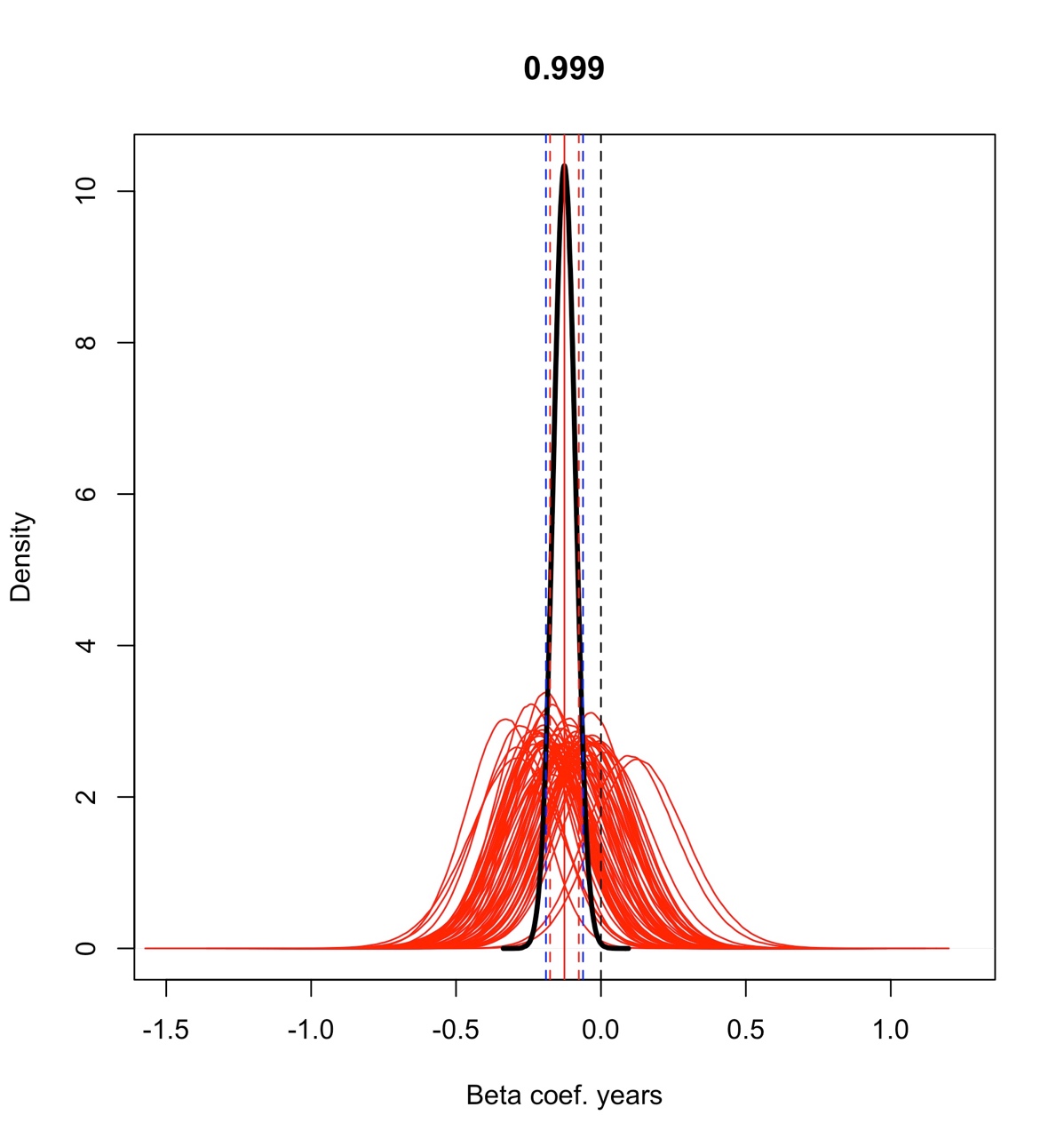
**

**Supplementary Figure S5.**


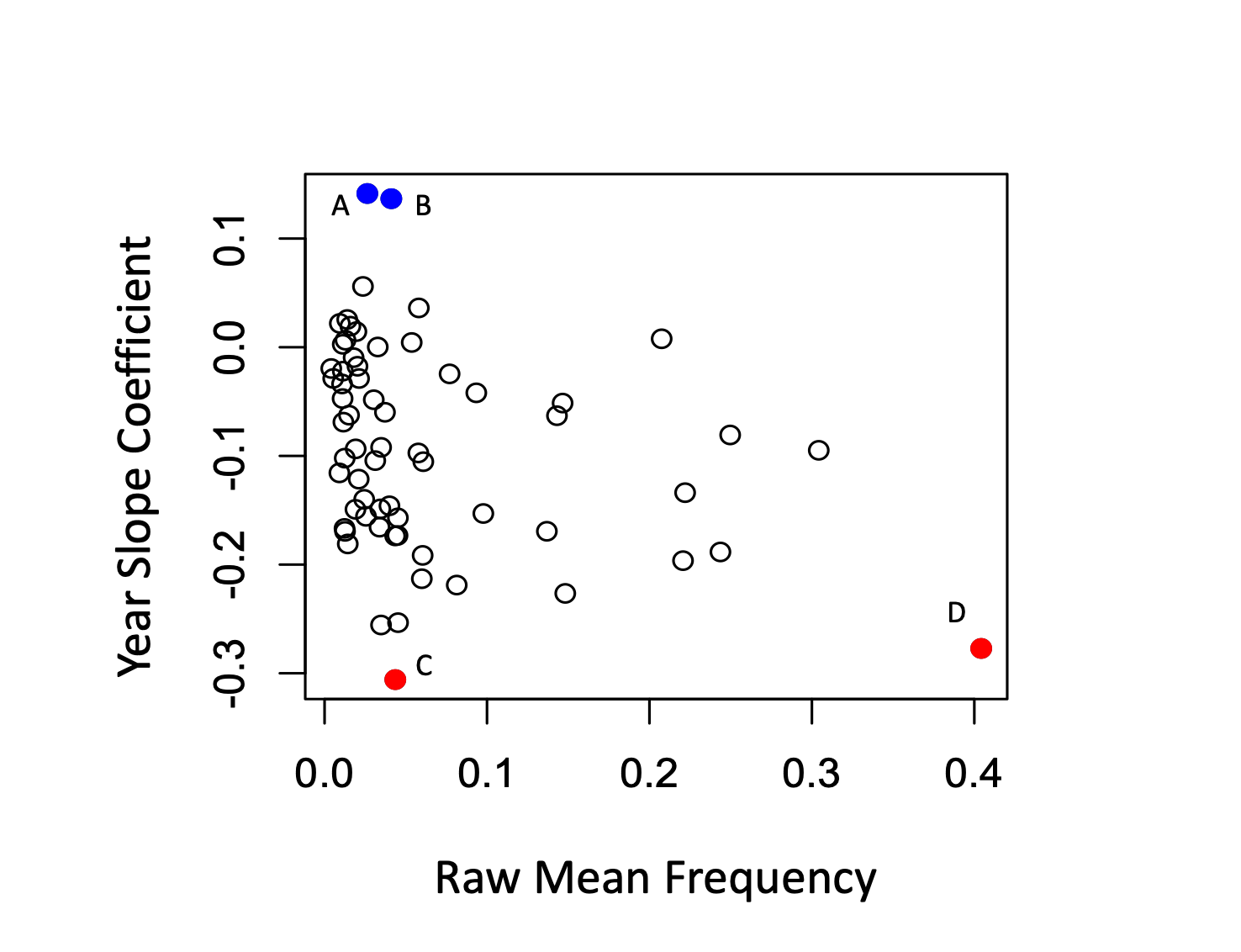


**Supplementary Figure S6.**

**
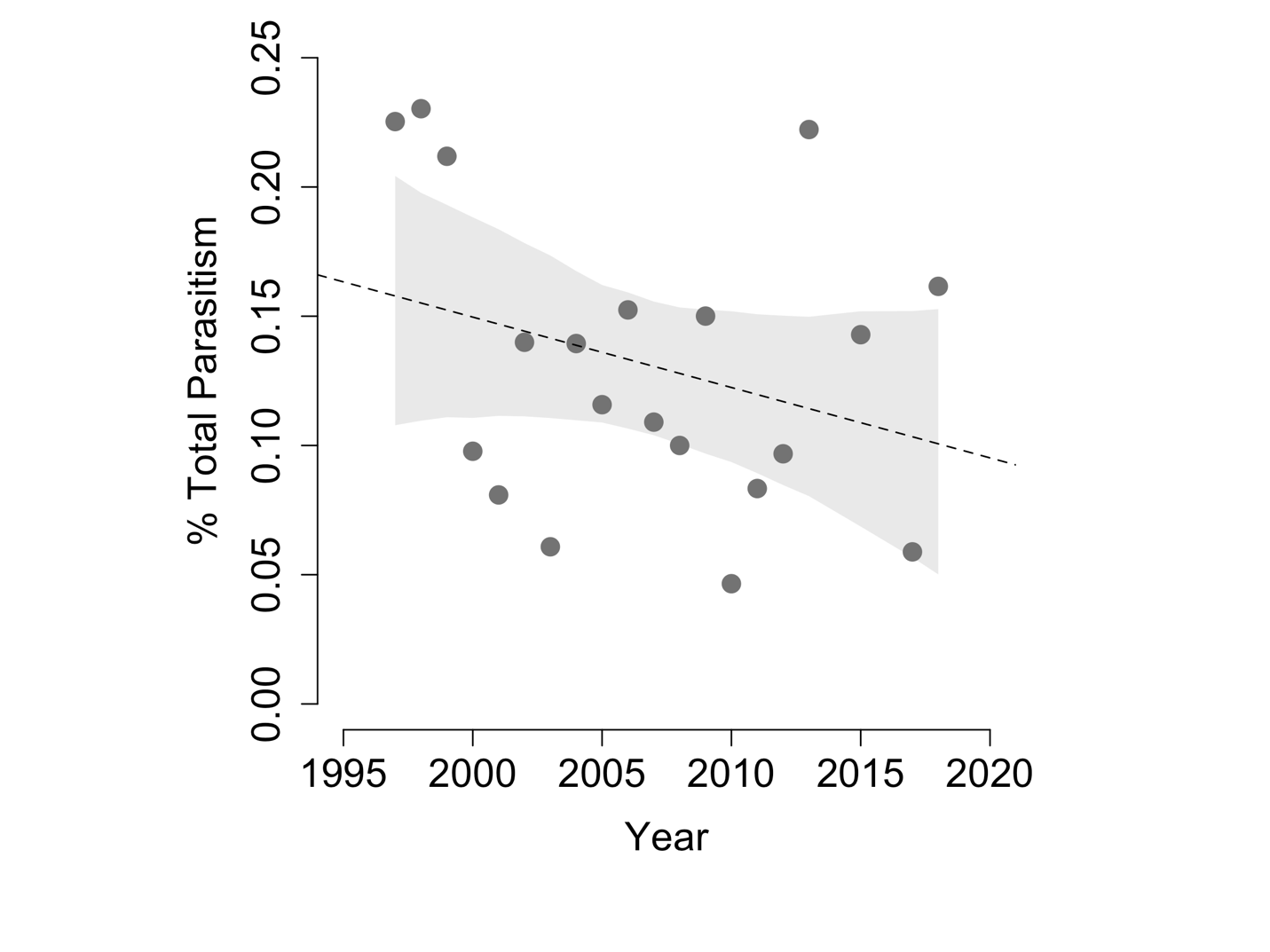
**

**Supplementary Figure S7.**

**
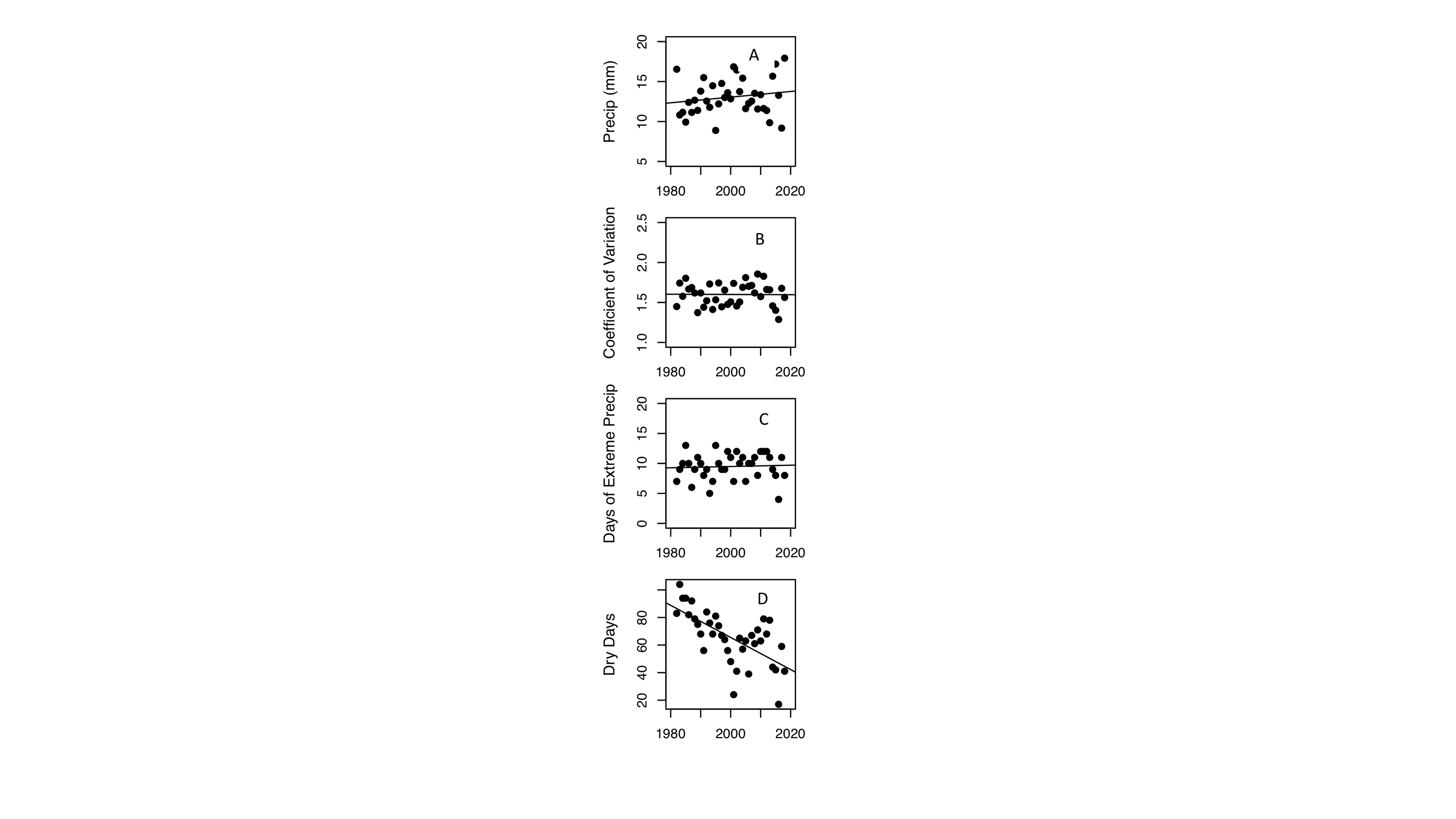
**

**Supplementary Figure S8.**

**
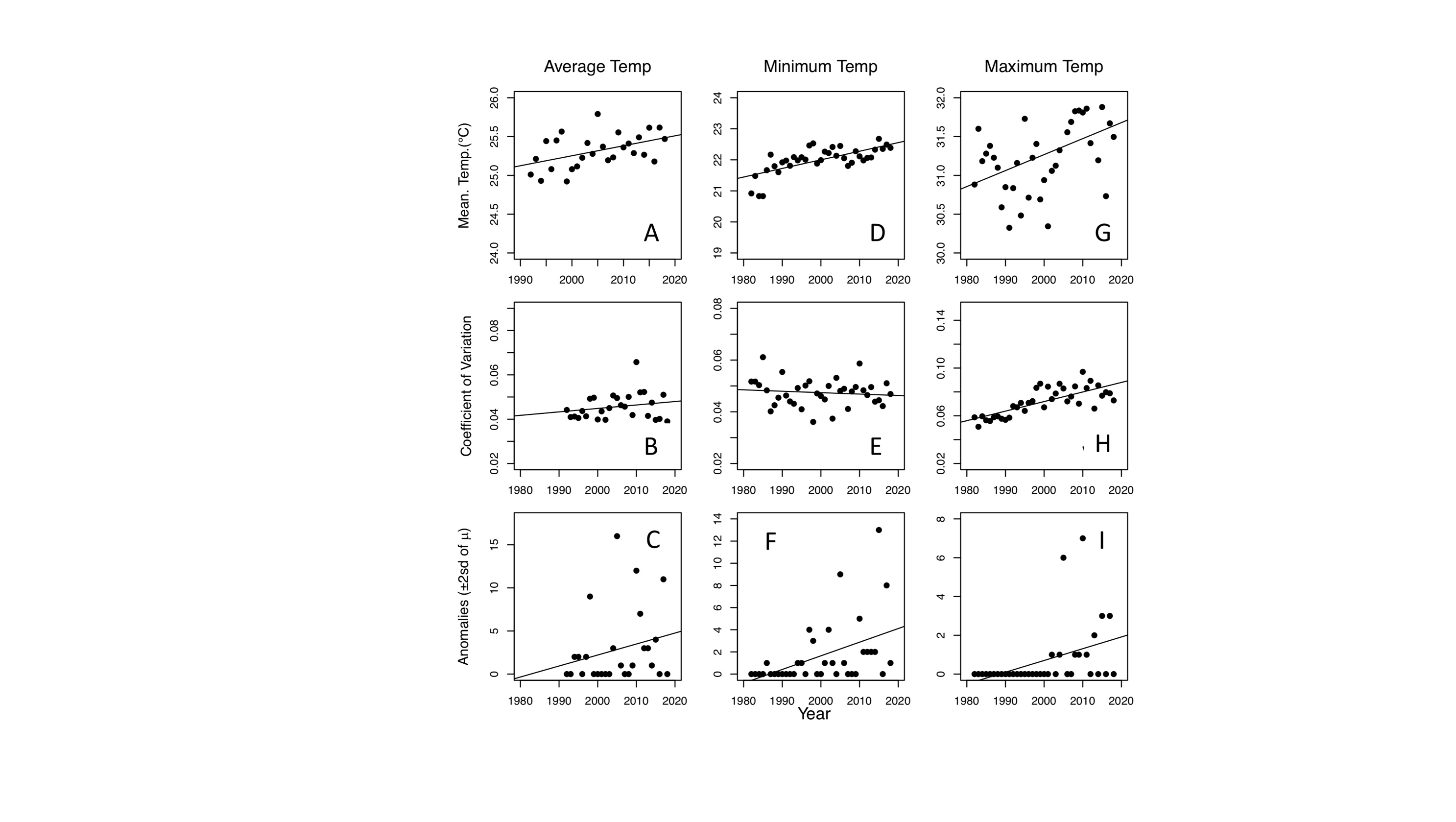
**

**Supplementary Figure S9.**

**
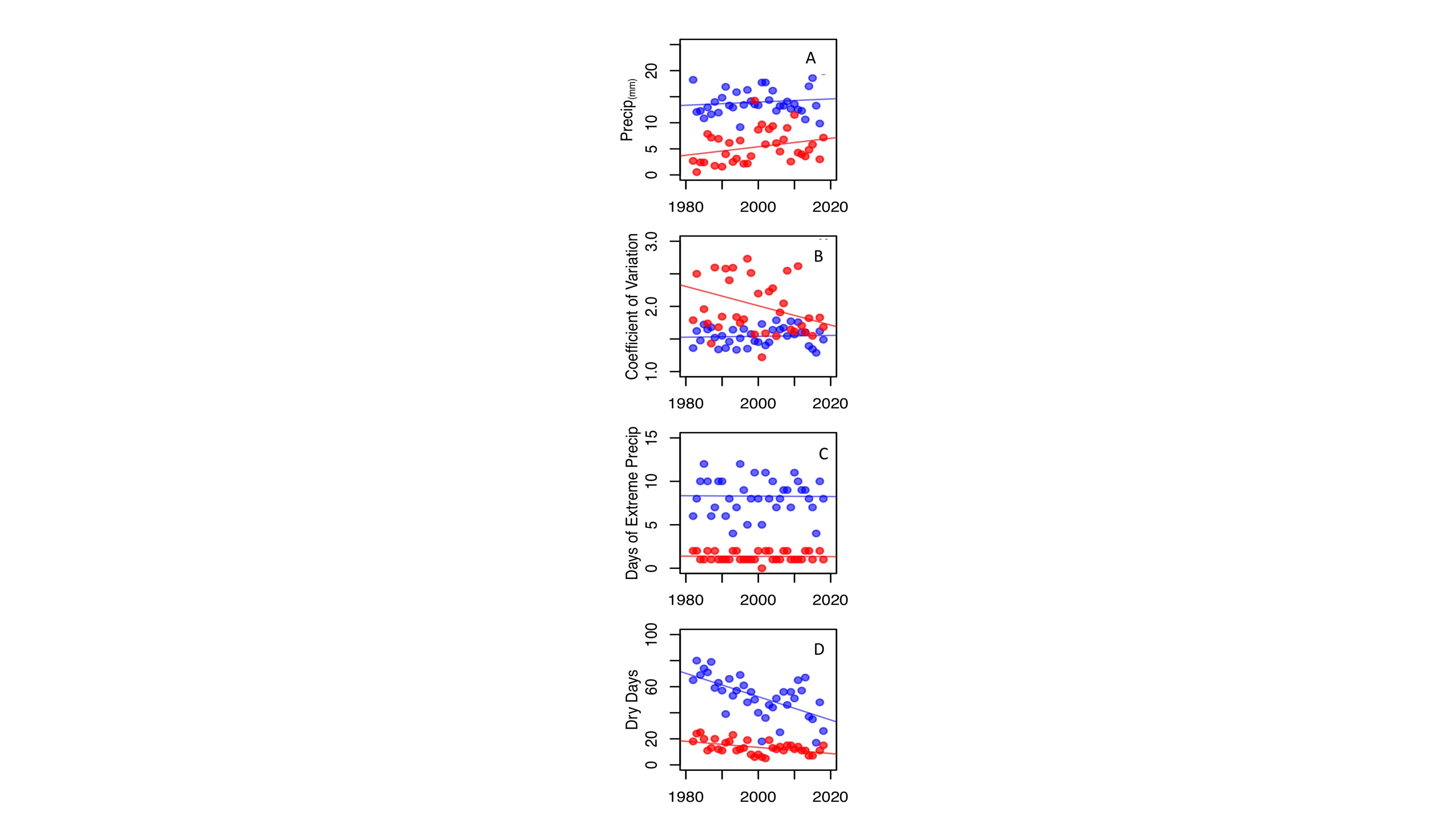
**

**Supplementary Figure S10.**

**
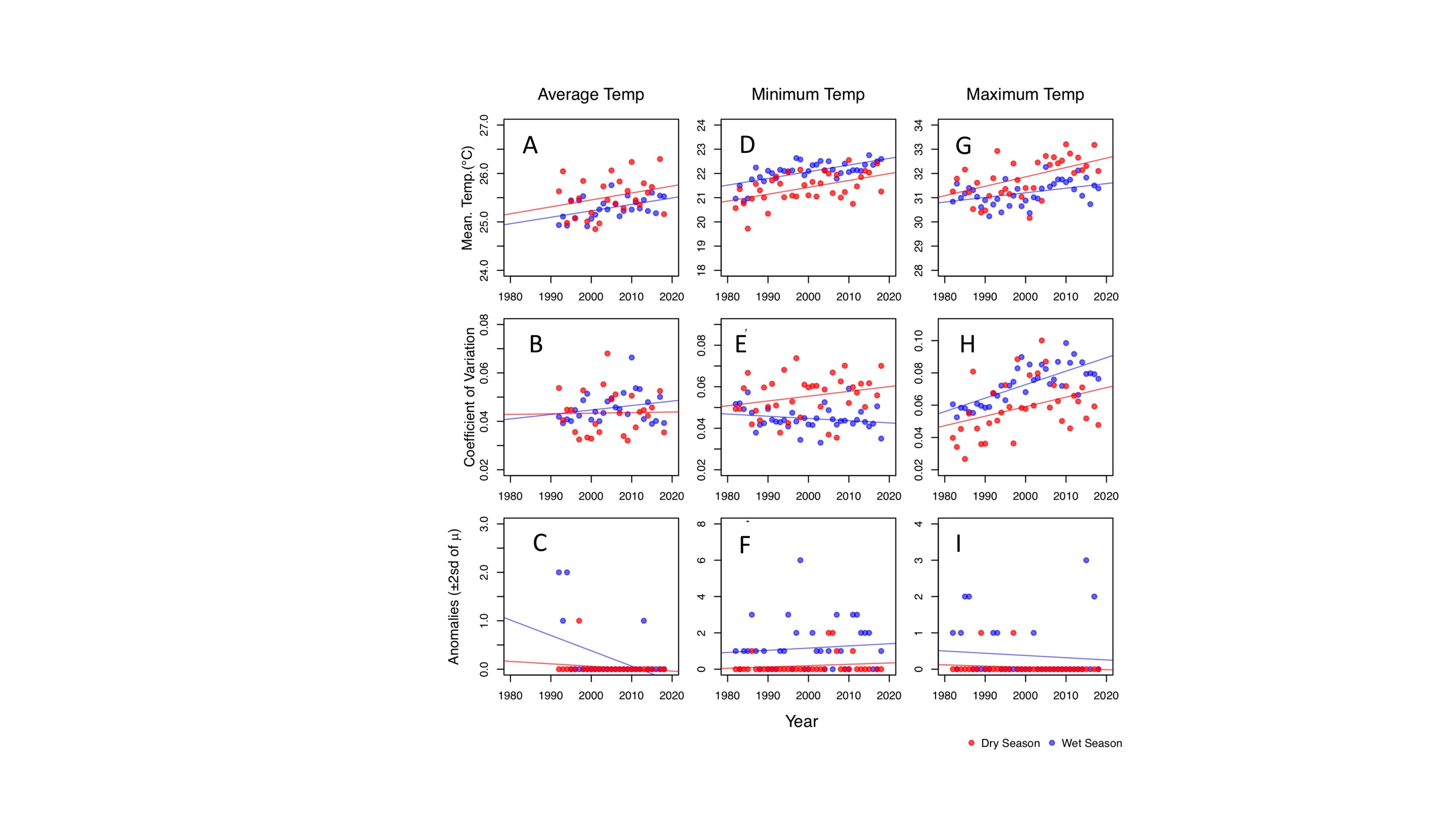
**

**Supplementary Figure S11.
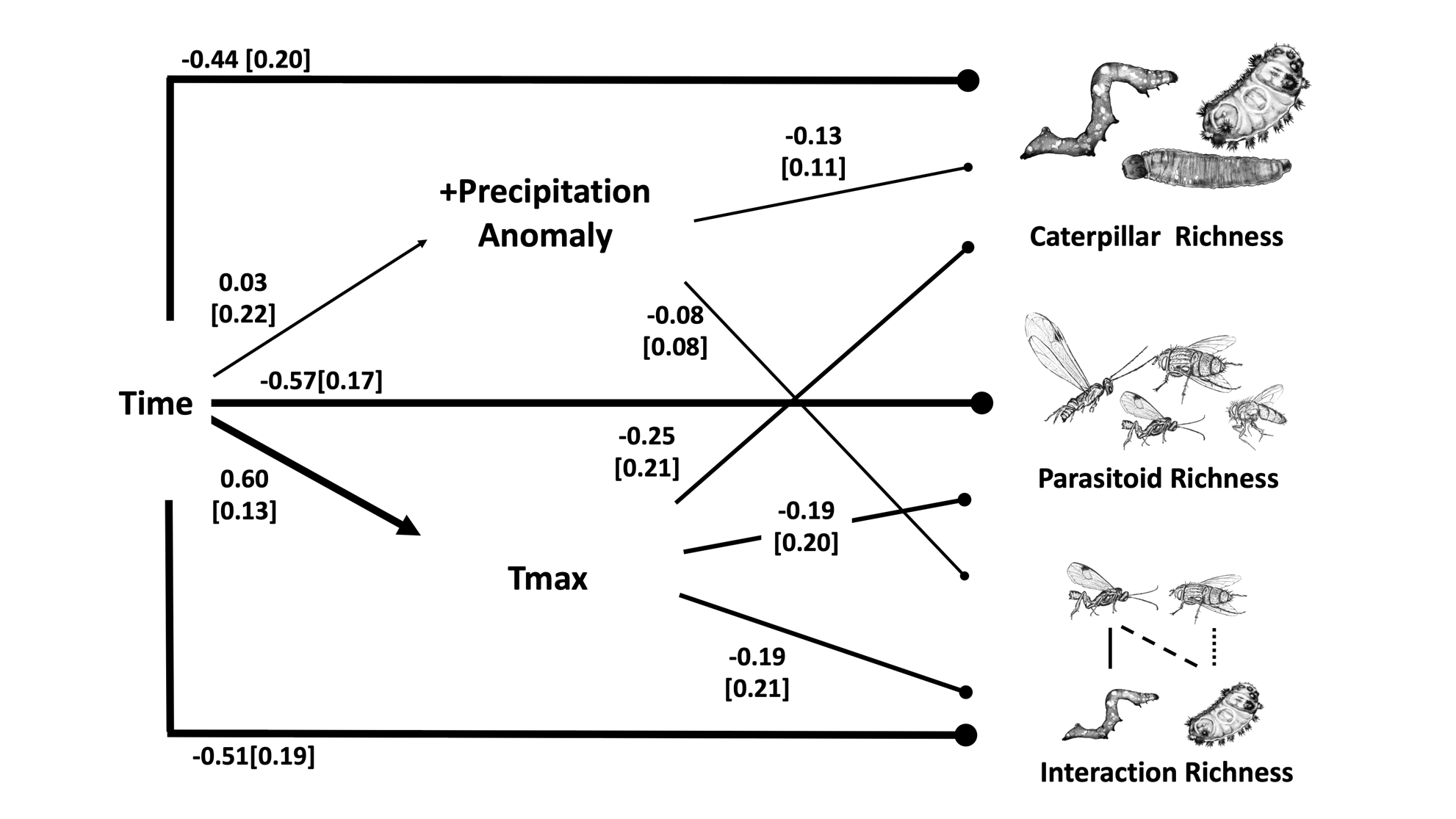
**

**Supplementary Fig. S12**

**
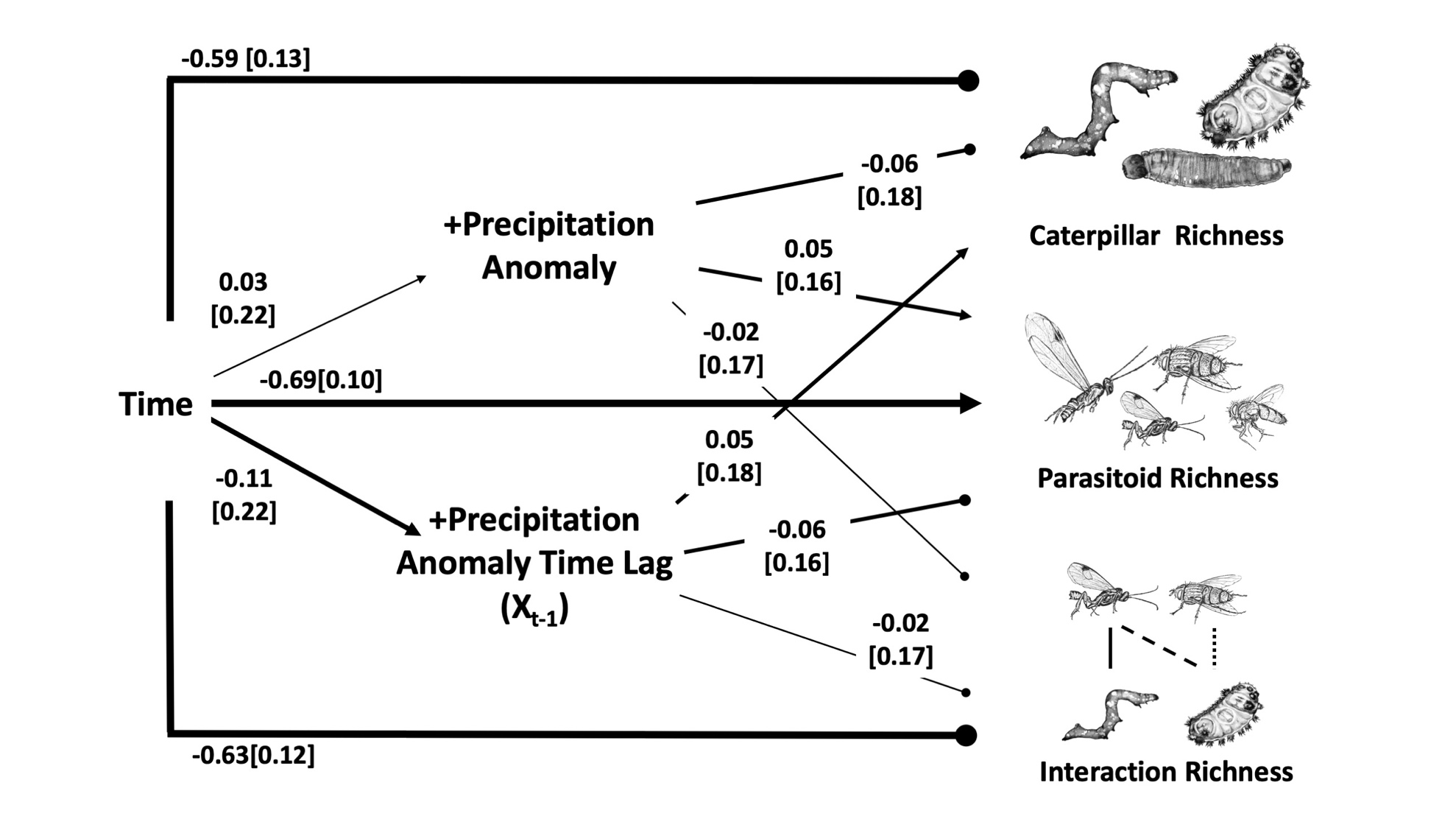
**

**Supplementary Table S1**

|  | **Standardized Coefficients** | | | | | |  | | **Unstandardized Coefficients** | | | | | |
| --- | --- | --- | --- | --- | --- | --- | --- | --- | --- | --- | --- | --- | --- | --- |
| **Lepidoptera Genera** | **Esitmate** | | **CI l_ower_** | | **CI_upper_** | | **Probability** | | **Estimate** | | **CI_Low_** | | **CI_High_** | |
| Saliana | 0.1412 | -0.0528 | | 0.3529 | | 0.1778 | | 0.0356 | | -0.0133 | | 0.0890 | |  |
| Eucereon | 0.1364 | -0.0579 | | 0.3485 | | 0.1866 | | 0.0344 | | -0.0146 | | 0.0879 | |  |
| Malocampa | 0.0561 | -0.1385 | | 0.2636 | | 0.3579 | | 0.0141 | | -0.0349 | | 0.0665 | |  |
| Achlyodes | 0.0363 | -0.1409 | | 0.2211 | | 0.3977 | | 0.0092 | | -0.0355 | | 0.0557 | |  |
| Memphis | 0.0255 | -0.1588 | | 0.2179 | | 0.4305 | | 0.0064 | | -0.0400 | | 0.0549 | |  |
| Zanola | 0.0219 | -0.1642 | | 0.2169 | | 0.4409 | | 0.0055 | | -0.0414 | | 0.0547 | |  |
| Cropia | 0.0193 | -0.1649 | | 0.2119 | | 0.4473 | | 0.0049 | | -0.0416 | | 0.0534 | |  |
| Dioptis | 0.0142 | -0.1678 | | 0.2035 | | 0.4604 | | 0.0036 | | -0.0423 | | 0.0513 | |  |
| Euptychia | 0.0077 | -0.1565 | | 0.1765 | | 0.4763 | | 0.0019 | | -0.0394 | | 0.0445 | |  |
| Pachydota | 0.0064 | -0.1886 | | 0.2104 | | 0.4835 | | 0.0016 | | -0.0476 | | 0.0531 | |  |
| Dunama | 0.0045 | -0.2107 | | 0.2329 | | 0.4893 | | 0.0011 | | -0.0531 | | 0.0587 | |  |
| Parides | 0.0027 | -0.1856 | | 0.1987 | | 0.4927 | | 0.0007 | | -0.0468 | | 0.0501 | |  |
| Scotura | 0.0002 | -0.1892 | | 0.1970 | | 0.4995 | | 0.0000 | | -0.0477 | | 0.0497 | |  |
| Telemiades | **-0.0096** | -0.1971 | | 0.1845 | | 0.5258 | | -0.0024 | | -0.0497 | | 0.0465 | |  |
| Anomis | **-0.0175** | -0.2028 | | 0.1734 | | 0.5478 | | -0.0044 | | -0.0511 | | 0.0437 | |  |
| Thracides | **-0.0193** | -0.2269 | | 0.1973 | | 0.5469 | | -0.0049 | | -0.0572 | | 0.0497 | |  |
| Megalopyge | **-0.0219** | -0.2072 | | 0.1692 | | 0.5599 | | -0.0055 | | -0.0522 | | 0.0427 | |  |
| Eueides | **-0.0247** | -0.2066 | | 0.1623 | | 0.5684 | | -0.0062 | | -0.0521 | | 0.0409 | |  |
| Isanthrene | **-0.0285** | -0.2090 | | 0.1562 | | 0.5800 | | -0.0072 | | -0.0527 | | 0.0394 | |  |
| Euclea | **-0.0287** | -0.2157 | | 0.1637 | | 0.5773 | | -0.0072 | | -0.0544 | | 0.0413 | |  |
| Protambulyx | **-0.0339** | -0.2400 | | 0.1800 | | 0.5825 | | -0.0085 | | -0.0605 | | 0.0454 | |  |
| Acharia | **-0.0420** | -0.2226 | | 0.1417 | | 0.6166 | | -0.0106 | | -0.0561 | | 0.0357 | |  |
| Temenis | **-0.0471** | -0.2533 | | 0.1646 | | 0.6147 | | -0.0119 | | -0.0639 | | 0.0415 | |  |
| Pericopis | **-0.0480** | -0.2601 | | 0.1702 | | 0.6137 | | -0.0121 | | -0.0656 | | 0.0429 | |  |
| Heliconius | **-0.0515** | -0.2312 | | 0.1313 | | 0.6429 | | -0.0130 | | -0.0583 | | 0.0331 | |  |
| Consul | **-0.0599** | -0.2490 | | 0.1324 | | 0.6574 | | -0.0151 | | -0.0628 | | 0.0334 | |  |
| Talides | **-0.0624** | -0.2479 | | 0.1260 | | 0.6667 | | -0.0157 | | -0.0625 | | 0.0318 | |  |
| Hypothyris | **-0.0632** | -0.2466 | | 0.1226 | | 0.6708 | | -0.0159 | | -0.0622 | | 0.0309 | |  |
| Tithraustes | **-0.0690** | -0.2756 | | 0.1408 | | 0.6657 | | -0.0174 | | -0.0695 | | 0.0355 | |  |
| Chlosyne | **-0.0808** | -0.2558 | | 0.0951 | | 0.7234 | | -0.0204 | | -0.0645 | | 0.0240 | |  |
| Hamadryas | **-0.0919** | -0.2877 | | 0.1040 | | 0.7281 | | -0.0232 | | -0.0726 | | 0.0262 | |  |
| Dubiella | **-0.0933** | -0.2933 | | 0.1070 | | 0.7268 | | -0.0235 | | -0.0740 | | 0.0270 | |  |
| Automeris | **-0.0946** | -0.2640 | | 0.0751 | | 0.7638 | | -0.0238 | | -0.0666 | | 0.0189 | |  |
| Gamelia | **-0.0975** | -0.2897 | | 0.0946 | | 0.7436 | | -0.0246 | | -0.0730 | | 0.0238 | |  |
| Epimecis | **-0.1017** | -0.2948 | | 0.0908 | | 0.7523 | | -0.0256 | | -0.0743 | | 0.0229 | |  |
| Phaeoblemma | **-0.1043** | -0.3068 | | 0.0977 | | 0.7475 | | -0.0263 | | -0.0774 | | 0.0246 | |  |
| Spodoptera | **-0.1052** | -0.2831 | | 0.0724 | | 0.7774 | | -0.0265 | | -0.0714 | | 0.0183 | |  |
| Hypercompe | **-0.1154** | -0.3246 | | 0.0915 | | 0.7638 | | -0.0291 | | -0.0818 | | 0.0231 | |  |
| Anacrusis | **-0.1211** | -0.3104 | | 0.0658 | | 0.7976 | | -0.0305 | | -0.0783 | | 0.0166 | |  |
| Quentalia | **-0.1337** | -0.3443 | | 0.0727 | | 0.7981 | | -0.0337 | | -0.0868 | | 0.0183 | |  |
| Olceclostera | **-0.1397** | -0.3259 | | 0.0428 | | 0.8374 | | -0.0352 | | -0.0822 | | 0.0108 | |  |
| Phoebis | **-0.1458** | -0.3630 | | 0.0654 | | 0.8126 | | -0.0368 | | -0.0915 | | 0.0165 | |  |
| Milanion | **-0.1488** | -0.3407 | | 0.0387 | | 0.8459 | | -0.0375 | | -0.0859 | | 0.0097 | |  |
| Antichloris | **-0.1490** | -0.3827 | | 0.0762 | | 0.8027 | | -0.0376 | | -0.0965 | | 0.0192 | |  |
| Cyclomia | **-0.1530** | -0.3144 | | 0.0062 | | **0.8910** | | -0.0386 | | -0.0793 | | 0.0016 | |  |
| Oraesia | **-0.1553** | -0.3526 | | 0.0368 | | **0.8505** | | -0.0392 | | -0.0889 | | 0.0093 | |  |
| Astraptes | **-0.1567** | -0.3104 | | -0.0055 | | **0.9080** | | -0.0395 | | -0.0783 | | -0.0014 | |  |
| Adelpha | **-0.1656** | -0.3466 | | 0.0110 | | **0.8852** | | -0.0418 | | -0.0874 | | 0.0028 | |  |
| Tigridia | **-0.1667** | -0.3600 | | 0.0210 | | **0.8725** | | -0.0420 | | -0.0908 | | 0.0053 | |  |
| Caligo | **-0.1693** | -0.3602 | | 0.0161 | | **0.8791** | | -0.0427 | | -0.0908 | | 0.0041 | |  |
| Myscelus | **-0.1695** | -0.3919 | | 0.0437 | | 0.8460 | | -0.0427 | | -0.0988 | | 0.0110 | |  |
| Desmia | **-0.1730** | -0.3511 | | 0.0007 | | **0.8992** | | -0.0436 | | -0.0885 | | 0.0002 | |  |
| Tarchon | **-0.1738** | -0.3430 | | -0.0078 | | **0.9102** | | -0.0438 | | -0.0865 | | -0.0020 | |  |
| Pachylia | **-0.1806** | -0.3751 | | 0.0073 | | **0.8909** | | -0.0455 | | -0.0946 | | 0.0018 | |  |
| Agaraea | **-0.1881** | -0.3765 | | -0.0056 | | **0.9067** | | -0.0474 | | -0.0949 | | -0.0014 | |  |
| Papilio | **-0.1915** | -0.3888 | | -0.0016 | | **0.9018** | | -0.0483 | | -0.0980 | | -0.0004 | |  |
| Dysschema | **-0.1961** | -0.3827 | | -0.0159 | | **0.9185** | | -0.0494 | | -0.0965 | | -0.0040 | |  |
| Apatelodes | **-0.2129** | -0.3964 | | -0.0364 | | **0.9391** | | -0.0537 | | -0.0999 | | -0.0092 | |  |
| Gonodonta | **-0.2186** | -0.3808 | | -0.0606 | | **0.9622** | | -0.0551 | | -0.0960 | | -0.0153 | |  |
| Hylesia | **-0.2266** | -0.4096 | | -0.0512 | | **0.9513** | | -0.0571 | | -0.1033 | | -0.0129 | |  |
| Pantographa | **-0.2532** | -0.4322 | | -0.0816 | | **0.9713** | | -0.0638 | | -0.1090 | | -0.0206 | |  |
| Dysodia | **-0.2554** | -0.4555 | | -0.0672 | | **0.9595** | | -0.0644 | | -0.1149 | | -0.0169 | |  |
| Emesis | **-0.2768** | -0.4933 | | -0.0768 | | **0.9630** | | -0.0698 | | -0.1244 | | -0.0194 | |  |
| Xylophanes | **-0.3059** | -0.4798 | | -0.1401 | | **0.9916** | | -0.0771 | | -0.1210 | | -0.0353 | |  |

**Supplementary Table S2.**

| **Network Property** | **1997-2001** | **2012-2018** |
| --- | --- | --- |
| **Node Richness** |  | |
| Host plant | 109 [325] | 79 [216] |
| Herbivore | 32[941] | 24 [257] |
| Parasitoid | 10 [385] | 2 [67] |
| Total | 151 [1651] | 105 [540] |
| **Link Richness** |  |  |
| Host Plant-Herbivore | 442 [1654] | 199 [409] |
| Herbivore-Parasitoid | 83 [547] | 19 [80] |
| Total | 525 [ 2201] | 218 [489] |
|  |  |  |

**Supplementary Table S3.**

|  | **Plant-Herbivore** | **Herbivore-Parasitoid** |
| --- | --- | --- |
| **β_wn_** | 0.92 | 0.81 |
| **β_ST_** | 0.27 | 0.68 |
| **β_OS_** | 0.64 | 0.14 |
| **β_S_** | 0.70 | 0.78 |

^1^ β calculated as Sorensen’s Index

**Supplementary Table S4.**

|  | Climate Variable | Estimate | Std.Error | P.Value | R.squared |
| --- | --- | --- | --- | --- | --- |
| Annual Mean | Precip (mm) | 0.036 | 0.035 | 0.315 | 0.029 |
|  | Tmin | 0.028 | 0.005 | 0.000 | 0.475 |
|  | Tmax | 0.021 | 0.007 | 0.005 | 0.200 |
|  | Avg. Temp. | 0.013 | 0.005 | 0.014 | 0.217 |
| Anomaly | Extreme Precip. Event | 0.011 | 0.034 | 0.749 | 0.003 |
|  | Drought Events | -1.165 | 0.229 | 0.000 | 0.426 |
|  | Tmin | 0.061 | 0.023 | 0.012 | 0.166 |
|  | Tmax | 0.123 | 0.040 | 0.004 | 0.211 |
|  | Avg. Temp. | 0.128 | 0.106 | 0.241 | 0.054 |
| Coefficient of Variation | Precip (mm) | 1.000 | 0.000 | 0.000 | 1.000 |
|  | Tmin | 0.000 | 0.002 | 0.950 | 0.000 |
|  | Tmax | 0.000 | 0.000 | 0.508 | 0.013 |
|  | Avg. Temp. | 0.001 | 0.000 | 0.000 | 0.559 |

**Supplementary Table S5**.

|  | Climate Variable | Estimate | Std. Error | P.value | R.squared |
| --- | --- | --- | --- | --- | --- |
| **Wet Season** |  |  |  |  |  |
| Annual Mean | Precip (mm) | 0.030 | 0.039 | 0.440 | 0.017 |
|  | Tmin | 0.028 | 0.005 | 0.000 | 0.452 |
|  | Tmax | 0.019 | 0.007 | 0.009 | 0.177 |
|  | Avg. Temp | 0.013 | 0.005 | 0.011 | 0.230 |
| Anomaly | Days of Extreme Precip. | -0.002 | 0.032 | 0.942 | 0.000 |
|  | Dry Days | -0.894 | 0.197 | 0.000 | 0.370 |
|  | Tmax | -0.006 | 0.012 | 0.605 | 0.008 |
|  | Tmin | 0.012 | 0.021 | 0.560 | 0.010 |
|  | Avg. Temp | -0.032 | 0.013 | 0.023 | 0.190 |
| Coefficient of Variation | Precip (mm) | 1.000 | 0.000 | 0.000 | 1.000 |
|  | Tmin | 0.001 | 0.002 | 0.760 | 0.003 |
|  | Tmax | 0.000 | 0.000 | 0.220 | 0.043 |
|  | Avg. Temp | 0.001 | 0.000 | 0.000 | 0.579 |
| **Dry Season** |  |  |  |  |  |
| Annual Mean | Precip (mm) | 0.081 | 0.049 | 0.102 | 0.077 |
|  | Tmin | 0.028 | 0.008 | 0.002 | 0.250 |
|  | Tmax | 0.039 | 0.012 | 0.002 | 0.248 |
|  | Avg. Temp | 0.014 | 0.010 | 0.165 | 0.079 |
| Anomaly | Days of Extreme Precip. | -0.001 | 0.009 | 0.881 | 0.001 |
|  | Dry Days | -0.234 | 0.072 | 0.003 | 0.235 |
|  | Tmax | -0.003 | 0.004 | 0.377 | 0.023 |
|  | Tmin | 0.007 | 0.008 | 0.385 | 0.022 |
|  | Avg. Temp | -0.005 | 0.005 | 0.331 | 0.039 |
| Coefficient of Variation | Precip (mm) | 1.000 | 0.000 | 0.000 | 1.000 |
|  | Tmin | -0.015 | 0.008 | 0.059 | 0.101 |
|  | Tmax | 0.001 | 0.000 | 0.007 | 0.201 |
|  | Avg. Temp | 0.000 | 0.000 | 0.702 | 0.006 |

**Supplementary Table S6**.

| **Year** | **Plant** | | **Caterpillar** | | | **Parasitoid** | | **Total Sampled** | | **Sample Effect/Hectare** | **Number of Volunteers per year** |
| --- | --- | --- | --- | --- | --- | --- | --- | --- | --- | --- | --- |
|  | **Family** | **Species** | | **Family** | **Species** | **Family** | **Species** | **Caterpillar** | **Parasitoid** |  |  |
| 1997 | 37 | 73 | | 19 | 111 | 6 | 51 | 726 | 64 | 234 | 22 |
| 1998 | 58 | 152 | | 27 | 261 | 8 | 114 | 1859 | 237 | 350 | 51 |
| 1999 | 56 | 159 | | 26 | 333 | 8 | 143 | 2531 | 273 | 270 | 31 |
| 2000 | 59 | 200 | | 28 | 406 | 7 | 115 | 2673 | 136 | 346 | 50 |
| 2001 | 59 | 136 | | 24 | 255 | 5 | 41 | 1603 | 48 | 306 | 40 |
| 2002 | 53 | 149 | | 24 | 234 | 6 | 70 | 2422 | 105 | 306 | 40 |
| 2003 | 46 | 118 | | 26 | 176 | 5 | 42 | 1820 | 51 | 318 | 43 |
| 2004 | 66 | 190 | | 24 | 198 | 6 | 78 | 2595 | 91 | 266 | 30 |
| 2005 | 55 | 143 | | 23 | 172 | 4 | 43 | 1662 | 84 | 266 | 30 |
| 2006 | 51 | 121 | | 17 | 82 | 4 | 22 | 699 | 41 | 146 | 1 |
| 2007 | 61 | 163 | | 21 | 163 | 6 | 39 | 1416 | 66 | 322 | 44 |
| 2008 | 55 | 169 | | 23 | 216 | 4 | 49 | 1747 | 65 | 186 | 10 |
| 2009 | 42 | 94 | | 23 | 126 | 5 | 23 | 647 | 43 | 146 | 10 |
| 2010 | 28 | 45 | | 16 | 64 | 3 | 5 | 289 | 3 | 146 | 1 |
| 2011 | 21 | 36 | | 16 | 43 | 3 | 4 | 146 | 4 | 182 | 9 |
| 2012 | 8 | 18 | | 11 | 17 | 2 | 3 | 152 | 2 | 182 | 9 |
| 2013 | 8 | 20 | | 12 | 21 | 3 | 4 | 73 | 4 | 146 | 9 |
| 2014 | 14 | 18 | | 12 | 20 | 3 | 3 | 31 | 2 | 174 | 7 |
| 2015 | 7 | 26 | | 12 | 20 | 4 | 6 | 120 | 4 | 238 | 23 |
| 2016 | 10 | 13 | | 8 | 11 | 3 | 3 | 24 | 3 | 198 | 13 |
| 2017 | 19 | 39 | | 17 | 40 | 2 | 7 | 117 | 1 | 198 | 13 |
| 2018 | 48 | 147 | | 24 | 153 | 3 | 41 | 685 | 30 | 146 | 13 |
